# 3D “Emboli” Culture Models Epithelial Breast Cancer Cell Oxidative Mitochondrial Metabolism with Relevance for Lung Metastasis

**DOI:** 10.1158/2767-9764.CRC-25-0587

**Published:** 2026-03-19

**Authors:** Kuppusamy Balamurugan, Melissa R. Mikolaj, Jonathan M. Weiss, Ronald J. Holewinski, Yu Fan, Shiba Prasad Dash, Xia Xu, Lois McKennett, Christopher W. Dell, Duncan Donohue, Ariana Vitale, Shashikala Ratnayake, Shikha Sharan, Qingrong Chen, Daoud Meerzaman, Thorkell Andresson, Daniel W. McVicar, Kedar Narayan, Esta Sterneck

**Affiliations:** 1 Laboratory of Cell and Developmental Signaling, Center for Cancer Research, National Cancer Institute, Frederick, Maryland.; 2 CCR Volume Electron Microscopy (CVEM), Center for Cancer Research, National Cancer Institute, Frederick, Maryland.; 3 Cancer Research Technology Program, Frederick National Laboratory for Cancer Research, Frederick, Maryland.; 4 Cancer Innovation Laboratory, Center for Cancer Research, National Cancer Institute, Frederick, Maryland.; 5 Protein Characterization Laboratory, Cancer Research Technology Program, Leidos Biomedical Research Inc., Frederick National Laboratory for Cancer Research, Frederick, Maryland.; 6 Computational Genomics and Bioinformatics Branch, Center for Biomedical Informatics and Information Technology, National Cancer Institute, National Institutes of Health, Rockville, Maryland.; 7 Laboratory Animal Sciences Program, Leidos Biomedical Research Inc., Frederick National Laboratory for Cancer Research, Frederick, Maryland.; 8 Statistical Consulting and Scientific Programming Group, Computer and Statistical Services, Data Management Services, Inc (a BRMI Company), National Cancer Institute, Frederick, Maryland.

## Abstract

Three-dimensional (3D) cell culture systems have emerged as powerful tools for modeling tumor biology in *ex vivo* settings. However, the diverse array of available 3D culture methods presents challenges in selecting the most appropriate model for specific research questions. This study provides a comparative analysis of breast cancer cells (SUM149, IBC-3, and MDA-MB-468) in the mammosphere culture (SphC) model or an “emboli” culture (EmC) model, which enrich for cancer stem cells and epithelial features, respectively. The EmC model, designed originally for inflammatory breast cancer, is characterized by media viscosity and mechanical rocking of the culture vessel. Notably, cells in EmC showed a distinct and durable reduction in cell proliferation *ex vivo* while demonstrating high capacity to establish experimental lung metastases *in vivo*. Ultrastructural quantitative analysis of electron microscopy images suggested that cells in EmC acquire nuclear and mitochondrial features that resemble those of tumor tissue. Proteomics, single-cell transcriptomics, and metabolic flux analyses showed that cells in EmC and SphC favor mitochondrial oxidative metabolism (OXPHOS) and glycolysis, respectively. EmC rendered cells hypersensitive to OXPHOS inhibition but more resistant to oxidative stress. Several genes associated with lung metastasis, including ID1, were specifically enriched in EmC. Given the emerging role of OXPHOS in breast cancer cell survival during dissemination and as established metastases, we propose that the EmC paradigm is a suitable *ex vivo* model to study signaling pathways relevant for basal epithelial breast cancer, including lung metastases, and to assess drug sensitivities and resistance mechanisms of such breast cancer cells *ex vivo*.

**Significance:**

This study provides an in-depth characterization of a resource-efficient, yet powerful 3D culture paradigm to improve the physiologic relevance of *ex vivo* approaches. Applicable to epithelial cancers, this model offers a platform to accelerate the discovery of physiologically relevant signaling pathways and specific cancer cell vulnerabilities.

## Introduction

For more than a century, cell culture has been a workhorse of biological research, conventionally conducted in two dimensions (1, 2). Although two-dimensional (2D) cultures provide ease of use and reproducibility, they are limited by nonphysiologic substrate stiffness and favor rapidly proliferating cell lines. To better recapitulate physiologic conditions, a variety of three-dimensional (3D) culture paradigms are being developed with varying degrees of complexity, including scaffolds or matrices, various cell types, and/or dynamic features (3–6). Each system presents distinct limitations and advantages depending on the research question to be addressed.

We previously demonstrated that metastatic breast cancer cells cultured in a liquid 3D paradigm, but not in 2D, recapitulate an *in vivo* signaling pathway that preserves E-cadherin protein expression even when its mRNA is downregulated (7). Epithelial–mesenchymal transition (EMT), a well-studied mechanism of metastasis, involves downregulation of genes for epithelial proteins like E-cadherin and induction of mesenchymal proteins such as vimentin. Driven by EMT transcription factors, it promotes stemness and invasiveness (8, 9). EMT gene signatures are notably upregulated in metastatic cancer (10) and contribute to the metastatic process (11, 12). However, most invasive ductal carcinomas and overt breast cancer metastases retain E-cadherin protein expression, and several studies have reported a positive correlation between E-cadherin, metastasis, and poor prognosis (13–18). Thus, an *ex vivo* metastatic breast cancer culture model that preserves E-cadherin–mediated cell–cell adhesions may provide a more physiologically relevant platform for studying metastatic biology and therapeutic vulnerabilities.

The 3D paradigm used in our prior study (7), originally developed by Lehman and colleagues (19) specifically to model intralymphatic emboli by inflammatory breast cancer (IBC) cells, incorporates lymph-like media viscosity and slow rocking for mild mechanical stimulation. We extended this emboli culture (EmC) model to additional breast cancer cell types, enabling the study of cell phenotypes under conditions in which cell–cell adhesions are maintained in all dimensions. Here, we present a systematic characterization of breast cancer cells cultured in EmC compared with 2D cultures, and in some contexts, with tumor tissue. Furthermore, because it is a widely used liquid 3D model, we included sphere culture (SphC), which was developed specifically to select for cancer stem cell (CSC) phenotypes (20, 21). Three basal epithelial and two mesenchymal cell lines were analyzed, all representing basal triple-negative breast cancer (TNBC), except one that is HER2+. Our results indicate that the 3D EmC promotes many features observed in epithelial primary tumors and lung metastases in breast cancer. Given the current urgency to increase research efforts with human *ex vivo* models, we propose EmC as a resource-efficient, yet powerful *ex vivo* approach to advance the identification of context-specific signaling pathways and actionable therapeutic vulnerabilities.

## Materials and Methods

### Cell culture and cell death assays

SUM149 (RRID: CVCL_3422), SUM159 (RRID: CVCL_5423), and MDA-IBC-3 (RRID: CVCL_HC47) cells originated from Asterand Bioscience and MDACC, respectively. MDA-MB-468 (RRID: CVCL_0419) and MCF-7 (RRID: CVCL_0031) cells were from the ATCC. MDA-MB-231-LM2 (RRID: CVCL_5998) and SUM190 RRID: CVCL_3423) cells were a kind gift from Dr. J. Massagué (Memorial Sloan Kettering Cancer Center) and Dr. P. Steeg (NCI), respectively. Cells were used at passages 2 to 30, last authenticated in 2025 by GenePrint10 (Promega), and routinely *Mycoplasma* tested by quantitative PCR (qPCR). SUM149 cells expressing luciferase were as described (7). Cells were cultured at 5% CO_2_/37°C in media with 10% FBS, 100 U/mL penicillin, and 100 μg/mL streptomycin as follows: MDA-MB-468, MDA-MB-231-LM2, and MCF-7 in Dulbecco’s Modified Eagle Medium (GIBCO #11965-092); SUM149, SUM190, and IBC-3 in Ham’s F-12 media (GIBCO #31765092) with 5 μg/mL hydrocortisone (MilliporeSigma #H-0135) and 1 μg/mL insulin (MilliporeSigma #I-0516); and SUM159 in RPMI with 2 mmol/L glutamine, 10 mmol/L HEPES, 1 mmol/L sodium pyruvate, 1X nonessential amino acids (GIBCO, #11140-050), and 55 μmol/L β-mercaptoethanol (GIBCO, #21985-023). In addition, all media contained 2.25% PEG8000 (MilliporeSigma #202452) except for SphC cultures, which were cultured in Mammocult media (StemCell Technologies #05620) supplemented with 2 μg/mL Heparin (StemCell Technologies #07980) and 0.44 μg/mL hydrocortisone. EmC was performed essentially as described (19) except that 6-well or 24-well ultra-low attachment dishes were used (Corning #3471, #3473). Briefly, 2.5 × 10^5^ cells were seeded in 6-well plates in 2 mL medium and gently rocked at approximately 40 rpm for 3 days unless otherwise indicated. Fresh medium (1 mL) was added on day 3 for longer culture periods. IACS-10759 (#S8731), sodium dichloroacetate (DCA; #S8615), and AGX51 (#E1152) were from Selleck Chemicals, FX-11 from CAYMAN Chemicals (#213971-34-7), and DMSO from MilliporeSigma (#D-2650).

To isolate the 3D cell assemblies, cells were centrifuged at 500 rpm for 30 seconds, washed with PBS, and treated with TrypLExpress (GIBCO #12604-013) for 10 minutes with intermittent pipetting before neutralization with cell culture medium. Cell numbers were counted by mixing 10 μL of cell suspension with 10 μL of Trypan Blue dye and analyzing by dye exclusion using a Countess II FL (Life Technologies). For cell proliferation measurements, about 2,000 cells were seeded into 96-well F-bottom black microplate (Griener #655090), and cell numbers were measured at the indicated times using a Celigo imaging cytometer (Nexcelom Biosciences). To assess the number of dying cells, cells were treated with 5 μg/mL propidium iodide (PI; MilliporeSigma #P4130) for 30 minutes and analyzed by PI-based cell death assay using a Celigo imaging cytometer (Nexcelom).

### Flow cytometry

For cell-cycle analysis, cells were cultured and trypsinized as described above. Ice-cold 70% ethanol (∼500 μL) was added slowly while vortexing the tubes, and the tubes were kept at 4°C for 2 hours. Cells were washed once with 1X FACS washing buffer (PBS + 0.1% BSA), and 200 μL of the PI staining solution (50 μg/mL PI + 3.8 mmol/L citrate buffer + 10 ng/mL RNase A) was added and incubated for overnight at 4°C. Samples were washed with FACS washing buffer and analyzed by flow cytometry. Analysis of CSC surface markers was performed essentially as described (22) using the following primary antibodies: CD44-PE (eBioscience# 12-0441-82) and CD24-FITC (eBioscience# 11-0247-41).

### Measurement of reactive oxygen species

For measurement of reactive oxygen species (ROS), 50,000 cells were resuspended in 400 μL serum-free medium, followed by incubation with CM-H2DCFDA (5 μmol/L; #6827, Thermo Fischer Scientific) for 20 minutes in the dark at 37°C. After 30 minutes, 400 μL ice-cold PBS + 0.5% BSA were added to stop the reaction, mixed well, and the cells were pelleted at 1,500 rpm for 5 minutes at room temperature (RT). The pellet was resuspended in 400 μL PBS + 0.5% BSA. To the DCF-DA preloaded cells, 100 μmol/L H_2_O_2_ was added and incubated for 15 minutes at RT in the dark. The cells were then pelleted (1,500 rpm, 5 minutes), resuspended in 200 μL PBS + 0.5% BSA, and transferred to a 96-well plate (Griener, #655090). The DCF fluorescence was measured using a Spectramax iD3 plate reader (Molecular Devices) using excitation (492 nm) and emission wavelengths (535 nm) settings.

### Seahorse Cell Mito Stress Test

Before the samples were loaded, the Seahorse 96-well microplate was coated with 30 μL of poly-L-lysine (MilliporeSigma #P4707) and kept inside the hood for 20 minutes. After the material was decanted, wells were washed twice with cell culture–grade water, incubated inside the hood for 30 minutes, and then placed in a non-CO_2_ incubator at 37°C for 30 minutes. This coated plate was taken for Seahorse Cell Mito Stress Test. The Seahorse XF Cell Mito Stress Test (cat. #103015-100, Seahorse Bioscience) uses modulators of respiration that target components of the electron transport chain in the mitochondria to measure key parameters of metabolic functions. The oxygen consumption rate (OCR) was measured using a Seahorse XF-96 analyzer. Before OCR measurement, the sensor cartridge was calibrated with calibration buffer at 37°C in a non-CO_2_ incubator overnight. A 72-hour culture was used unless indicated otherwise. For drug treatments, drugs were added 12 hours before analysis. Cultures were isolated, trypsinized for 5 minutes using TRYPLExpress, counted, and 200,000 cells were seeded onto the 96-well microplate coated with poly-L-lysine, washed with XF assay medium (pH 7.4), supplemented with 1 mmol/L pyruvate, 2 mmol/L glutamine, and 10 mmol/L glucose, and placed in a 37°C incubator without CO_2_ for 1 hour before calibration. One μmol/L oligomycin, 2 μmol/L protonophoric uncoupler (FCCP), and 0.5 μmol/L antimycin A + rotenone were preloaded in reagent ports A, B, and C. Three readings were taken after the addition of each reagent prior to injection with subsequent reagents. The OCR and extracellular acidification rate (ECAR) under the same conditions were automatically calculated and recorded by the sensor cartridge and Seahorse XF-96 software. The numerical data of samples in the test were normalized to the protein amount in each well.

### Protein isolation and Western blot analysis

Cells were lysed for 15 minutes on ice with cell lysis buffer containing 10 mmol/L Tris, 1 mmol/L EDTA, 400 mmol/L NaCl, 0.1% NP-40, 10 μL/mL of phosphatase inhibitors 2 and 3, and 10 μL/mL of protease inhibitor cocktail (MilliporeSigma #P8340). Samples were centrifuged at 12,700 rpm for 15 minutes at 4°C. Protein was quantified using Bradford assay kit (Bio-Rad #5000001), and 20 μg was separated on SDS-PAGE and transferred to nitrocellulose membranes. After blocking with 5% nonfat dry milk solution for 1 hour, the membrane was incubated with the indicated antibodies overnight. Following three washes with TBST (TBS containing 0.05% Tween-20), appropriate secondary antibody was added for 1 hour, followed by three washes before scanning with the iBRIGHT 1500 Imaging System (Thermo Fischer Scientific). Antibodies were obtained from the following sources: Cell Signaling Technology (ID1, #23369S; ID3, #9837: RRID AB_2732885; E-cadherin, #3195; and vimentin, #46173) and DHSB (A-actin, # 12G10).

### Xenograft tumors and experimental metastasis assays

To generate primary tumors, SUM149-GFP-Luc cells (2 × 10^6^; ref. 7) were orthotopically injected into NOD/SCID gamma (NSG) mice (RRID: BCBC_4611; 8–12 weeks old), and tumors at end point of about 2,000 mm^3^ tumor volume were collected and frozen for proteomics. For electron microscopy and array tomography, a small tumor of about 60 mm^3^ was collected and fixed immediately (for details, see Sample preparation for array tomography). For experimental lung metastases, mice were randomized to groups by weight, and about 0.5 million cells in 100 μL PBS were injected by the tail vein into 8- to 12-week-old NSG female mice. Within 2 hours, bioluminescence was determined to assure successful inoculation (0-day measurement). Bioluminescence was monitored at 2-week intervals for up to 20 weeks using the IVIS Spectrum imager (PerkinElmer, Inc.). At the end point, the lungs were inflated with fixative and paraffin-embedded, and sections stained with hematoxylin and eosin for histologic evaluation. Immunohistochemistry was performed according to standard procedures with primary antibodies for ID1 at 1:50 (Cell Signaling Technology #23369), Ki67 at 1:200 (Cell Signaling Technology #9027), or isotype control rabbit monoclonal IgG (Cell Signaling Technology # 3900).

All experiments were according to protocols approved by an Institutional Animal Care and Use Committee. Animal care was provided in accordance with the procedures outlined in the “Guide for Care and Use of Laboratory Animals,” including those pertaining to studies of neoplasia (National Research Council, 1996; National Academy Press; Washington, D.C.). NCI-Frederick is accredited by Association for Assessment and Accreditation of Laboratory Animal Care International and follows the Public Health Service Policy for the Care and Use of Laboratory Animals.

### Sample preparation for proteomic analysis

#### Tissue/cell lysis, digestion, and TMTpro labeling

Three-day cultures from 2D, EmC, and SphC from SUM149 cells were harvested and washed with PBS (2X), and the pellets and/or tissue samples were treated with 300 μL of EasyPep lysis buffer (Thermo Fischer Scientific #A45735) and 1 μL of universal nuclease (Thermo Fischer Scientific #88700). The samples were homogenized with pestle and centrifuged for 5 minutes at 14,000 *g*, and the supernatant was removed to a clean tube. Cell pellets were treated with 100 μL of EasyPep lysis buffer and 1 μL of universal nuclease. Protein concentration was determined using the bicinchoninic acid method. For each sample, the volume corresponding to 50 μg of protein was adjusted to 100 μL total with lysis buffer, treated with 50 μL of reducing and alkylating solutions provided with the EasyPep kit (Thermo Fischer Scientific #A40006), incubated at 95°C for 10 minutes, and then allowed to cool to RT. Samples were then treated with 4 μg of trypsin/LysC provided with the EasyPep kit and incubated at 37°C for 19 hours overnight. Samples were then treated with 40 μL of 12.5 μg/μL TMTpro in acetonitrile and incubated at RT for 1 hours. Samples were quenched with 50 μL of 5% hydroxylamine and 20% formic acid (FA) for 10 minutes and then combined. Samples were cleaned using spin column provided with the EasyPep kit, and eluted peptides were dried in a SpeedVac.

#### Liquid chromatography–Mass spectrometry analysis

Dried peptides were resuspended in 0.1% FA and injected in triplicate using a Dionex U3000 RSLC in front of an Orbitrap Eclipse (Thermo Fischer Scientific) equipped with an EasySpray ion source. Solvent A consisted of 0.1% FA in water and solvent B consisted of 0.1% FA in 80% ACN. Loading pump consisted of solvent A and was operated at 7 μL/minute for the first 6 minutes of the run and then dropped to 2 μL/minute when the valve was switched to bring the trap column (Acclaim PepMap 100 C18 HPLC Column, 3 μm, 75 μm I.D., 2 cm, PN 164535, Thermo Fischer Scientific) in-line with the analytic column (EasySpray, 2 μm, 75 μm I.D., 50 cm, PN ES903, Thermo Fischer Scientific). The gradient pump was operated at a flow rate of 300 nL/minute, and each run used a linear liquid chromatography (LC) gradient of 5% to 7% B for 1 minute, 7% to 30% B for 134 minutes, 35% to 50% B for 35 minutes, 50% to 95% B for 4 minutes, holding at 95% B for 7 minutes, and then re-equilibration of the analytic column at 5% B for 17 minutes. All mass spectrometry (MS) injections used the TopSpeed method with a 3 seconds cycle time that consisted of the following: spray voltage of 1,800 V and ion transfer temperature of 275°C. MS1 scans were acquired in the Orbitrap with resolution of 120,000, AGC of 4e5 ions, maximum injection time of 50 milliseconds, and mass range of 400 to 1,600 m/z; MS2 scans were acquired in the ion trap using the turbo scan method with AGC of 1e4, maximum injection time of 35 milliseconds, CID energy of 35%, isolation width of 0.7 Da, intensity threshold of 1e4, and charges 2 to 5 for MS2 selection. Real-time search (RTS) was enabled for TMTpro-labeled peptides using the UniProt Human Feb 2020 database. RTS settings were full tryptic peptides, 1 max missed cleavage, fixed carbamidomethyl on Cys, fixed TMTpro on Lys and peptide N-terminus, and variable oxidation on Met. MS3 scans for peptides identified by RTS were acquired in the Orbitrap with resolution of 50,000, AGC of 2.5e5, maximum injection of 86 milliseconds, mass range 100 to 500 m/z, and isolation width of 2 Da. Advanced peak determination, monoisotopic precursor selection, and EASY-IC for internal calibration were enabled, and dynamic exclusion was set to a count of 1 for 15 seconds.

#### Database searching and postprocessing

All three injections were batched together as fractions, and all MS files were searched with Proteome Discoverer 2.3 (RRID: SCR_014477) using the Sequest node. Data was searched against the UniProt (RRID: SCR_002380) Human database from Feb 2020 using a full tryptic digest, 2 maximum missed cleavages, minimum peptide length of 6 amino acids and maximum peptide length of 144 amino acids, an MS1 mass tolerance of 10 ppm, MS2 mass tolerance of 0.6 Da, variable oxidation on methionine (+15.995 Da), and fixed modifications of carbamidomethyl on cysteine (+57.021), TMTpro (+304.207) on lysine, and peptide N-terminus. Percolator (RRID: SCR_005040) was used for FDR analysis, and TMTpro reporter ions were quantified using the MS3 Reporter Ion Quantifier node and normalized on the total peptide intensity of each channel. TMTpro channel assignment for conditions can be found in Supplementary File S5. Statistical analysis was performed within PD2.3, log_2_ fold change (FC) was calculated based on the median of each group, and adjusted *P* value was calculated using ANOVA. Differentially expressed proteins (DEP) were set using a log_2_ FC cutoff of ± 0.6 and adjusted *P* value of < 0.05. For proteins that were observed in two or more replicates of a condition and 0 of the other in a comparison, the log_2_ FC was set to ± 0.1 the highest/lowest value in the comparison, and the adjusted *P* value was set slightly lower than the lowest value calculated in the comparison. Pathway enrichment analysis was performed using Ingenuity Pathway Analysis (QIAGEN; RRID: SCR_008653). The log_2_ FC threshold was lowered to ± 0.1, and the adjusted *P* value was kept at <0.05. Data have been deposited at MassIVE (https://massive.ucsd.edu/; RRID: SCR_013665) with accession number MSV000099222.

### Reversed-phase ion-pairing LC-MS^2^ assay for measuring cell central carbon metabolites

Three-day cultures of SUM149 and IBC-3 cell under 2D, SusC, EmC, and SphC conditions were used. All targeted central carbon metabolite (CCM) reference compounds, including acetyl-CoA and α-ketoglutarate, were purchased from Sigma-Aldrich. The stable isotope-labeled internal standards (SI-CCM) were ^13^C_3_-lactate and ^13^C_4_-succinic acid (Cambridge Isotope Laboratory), as well as ^13^C_6_-glucose-6-phosphate and ^13^C_6_-fructose-1,6-diphosphate (Medical Isotopes, Inc.). All CCM and SI-CCM analytic standards have reported chemical and isotopic purity ≥98% and were used without further purification. OmniSolv LC-MS–grade acetonitrile and methanol were obtained from EMD Millipore. Tributylamine, LC-MS–grade acetic acid and FA were purchased from Thermo Fisher Scientific. LC-MS–grade ammonium formate was obtained from CovaChem. All chemicals and solvents used in this study were HPLC- or reagent-grade unless otherwise noted. The isotope dilution LC/MS-MS method adapted from previous publications (23, 24) was used to measure the concentrations of cell CCMs. In brief, cell pellets were extracted with chilled 80% methanol in water supplemented with appropriate isotopic standards. Reversed-phase ion-pairing LC-MS^2^ analysis was performed using the Thermo TSQ Quantiva triple quadrupole mass spectrometer (Thermo Fischer Scientific) coupled with the Shimadzu 20AC-XR LC system for the CCM measurement. The mass spectrometers were operated in negative ion mode and set to monitor parent-product ion transitions using selected reaction monitoring. Quantitation of targeted metabolites was performed using Xcalibur Quan Browser (Thermo Fischer Scientific). Calibration curves for each metabolite were constructed by plotting CCM/SI-CCM peak area ratios obtained from the calibration samples versus their CCM concentrations and fitting the data using linear regression with 1/*X* weighting. The analyte concentrations in experimental samples were then interpolated using the linear function obtained from the calibration curve.

### RNA isolation and quantitative RT-PCR

Total RNA was isolated using GeneJET RNA Purification Kit (Thermo Fischer Scientific, #K0732), and 2 μg RNA was taken for cDNA synthesis using Superscript Reverse Transcriptase III (RT) according to the manufacturer’s instructions (Invitrogen, #18080044). qPCR was carried out with Fast SYBR Green master mix (#4385612, Applied Biosystems) using the 7500 Fast Real-Time PCR instrument (Applied Biosystems), and the relative expression levels were measured using the relative quantitation ΔΔ*C*t method and normalized to *actin*. Data represent three independent biological replicates, each measured as technical triplicates. Primers were as follows: *ID1* (5′-aaa​cgt​gct​gct​cta​cga​ca-3′ and 5′-gat​tcc​gag​ttc​agc​tcc​aa-3′; *MRPS12* (5′-gct​acc​tgc​tcc​atg​gct​ac-3′ and 5′-cac​ttg​cga​ttg​gct​gag​t-3′); *NDUSF6* (5′-cac​act​ggc​cag​gtt​tat​ga-3′ and 5′-tgg​tgc​tgt​ctg​aac​tgg​ag-3′); and *RPLP0* (5′-gca​atg​ttg​cca​gtg​tct​gtc-3′ and 5′-gcc​ttg​acc​ttt​tca​gca​agt-3′).

### Single-cell library preparation, sequencing, and analysis method

Three-day cultures of SUM149 and IBC-3 cells grown under 2D, EmC, and SphC conditions were harvested, washed with PBS, and had RNA isolated as per the manufacturer’s instructions, and 2 μg RNA was taken. Two biological replicates were labeled with Hashtag1 or Hashtag2 for each experimental condition. The single-cell gene expression libraries were generated using the 10x Chromium Single Cell 3′ Reagent Kit v3 (CG000183 Rev A) with feature barcode technology in accordance with the guidelines provided by 10X Genomics. The TotalSeq -B anti-human Hashtag reagents (BioLegend #394602, 394604, 394606) were used for cell hashing and multiplex samples. These libraries were sequenced on a NovaSeq system utilizing two SP flowcells from Illumina. The sequencing run was setup as 28 cycles for read 1, 8 cycles for i7 index, and 75 cycles for read 2 according to protocol recommendation. Sequencing run demultiplexing was performed by using Cellranger mkfastq, with one mismatch allowed in the barcodes. Cell Ranger v4.0.0 was applied for alignment, tagging, and gene and transcript counting for Chromium 3′ gene expression libraries, and sample demultiplexing was done using Cell Ranger v4.0.0 based on hashtag feature barcodes. The human genome GRCH38 reference with Gencode v32/Ensembl98 annotation (refdata-gex-GRCh38-2020-A; RRID: SCR_014966) were used as reference in Cell Ranger analysis. Seurat (v5; ref. 25) R package was used for single-cell RNA-seq (scRNA-seq) data analyses, including cell hashtag filtering, quality control, normalization, clustering, visualization, as well as differential expression (DE) analysis. After cell hashtag demultiplexing, only singlets with reasonable expression matrices were kept for downstream analysis. For DE analysis, we conducted pairwise comparisons among the three culture conditions, and we also compared each culture condition versus the other two conditions. Differentially expressed genes (DEG) were identified with adjusted *P* value less than 0.05 and average log_2_ FC more than 1 (or less than −1) cutoffs. Genes commonly upregulated or downregulated in both cell lines were used as inputs for QIAGEN Ingenuity Pathway Analysis (QIAGEN, Inc., https://digitalinsights.qiagen.com/IPA, version 01-23-01; RRID: SCR_008653; ref. 26). Data are available at Gene Expression Omnibus (GEO) under accession number GSE309277.

### Sample preparation for electron microscopy

SUM149 breast cancer cells were cultured as described above, harvested, and washed once with PBS, and Karnovsky’s fixative (4% formaldehyde and 2% glutaraldehyde in 0.1 mol/L cacodylate buffer) was added and incubated at RT for 5 hours with gentle pipetting every hour to prevent clumping. Samples were centrifuged at 500 rpm for 30 seconds, and the supernatant was decanted. One mL cacodylate buffer (0.1 mol/L) was added and centrifuged again at 500 rpm for 30 seconds. The pellet was suspended in cacodylate buffer and kept at 4°C. Cells from 2D culture were pelleted and embedded in 1% low-melt agarose before continuing with sample preparation. All other cell growth types were left free-floating. Cells were post-fixed in 2% osmium tetroxide and 1.5% potassium ferricyanide in 0.1 mol/L sodium cacodylate for 1 hour at RT, washed with ultrapure water, and then stained with 1% aqueous uranyl acetate overnight at 4°C. After further washes in ddH_2_O, the cells were treated with lead aspartate at 65°C for 30 minutes and washed again. The samples were dehydrated through graded ethanol (35%, 50%, 70%, 95%, and 100% × 5, 10 minutes each) and propylene oxide (PO; 5 × 10 minutes). The cells were infiltrated with increasing concentrations of Polybed 812 resin (hard formulation) in PO (resin: PO; 1:3, 1:2, 3:1, and 100%), embedded in 100% degassed resin, and cured at 65°C for 48 hours in flat BEEM capsules (EMS, Warrington). After polymerization, resin blocks were trimmed with a Leica EM TRIM2 milling system. After trimming the block face down, 100-nm sections were cut with a 45 degrees ultra-diamond knife (Diatome) using a Leica ARTOS ultramicrotome. Sections were collected with a loop and placed on glow-discharged ITO coverslips.

### Array tomography SEM imaging and segmentation

Sections on ITO coverslips were mounted onto 4-inch type-p silicon wafers using copper tape and affixed to a 4-inch stage-decel holder in the GeminiSEM 450 (Carl Zeiss). Low-resolution overview scans of individual resin sections were acquired at 150 nm pixel resolution using ATLAS 5 Array Tomography software (Fibics). High-resolution (5 nm pixels) large areas were imaged by stitching multiple fields, enabled by automated stage movement and a four-quadrant backscatter detector (aBSD, Zeiss). Image strips of roughly 40,000 × 8,000 pixels were cropped from the larger images to capture roughly similar numbers of cells. These strips were imported into Napari (https://napari.org/stable/) in which segmentation was performed using the empanada plugin. The appropriate deep learning models were deployed in the 2D inference module with image tiling: mitochondria were segmented using MitoNet_v1 (27) lipid droplets (LD) with DropNet_base_v1, and nuclei with NucleoNet_base_v1 (to be reported elsewhere). Both models are prepackaged with our open-source napari plugin empanada v1.2. The full code base for empanada-napari can be found at https://github.com/volume-em/empanada-napari. Errors in model predications were corrected manually in empanada-napari. To facilitate correction in these large images, the “create tiles” module in empanada was used to generate smaller image tiles to reduce the time needed for manual adjustments. After all corrections were completed on the individual tiles, both the images and their segmentations were reassembled using the “merge tiles” module in empanada. Cell segmentation was performed in 3DSlicer Ver. 5.8.1 (28) using thresholding to delineate borders for each cell in every image, followed by filling in using the fill tool in Napari. Quantitative measurements of cells, mitochondria, LDs, and nuclei were obtained with the napari-clusters-plotter plugin at “https://github.com/haesleinhuepf/napari-skimage-regionprops”, which utilizes region props from scikit-image (https://doi.org/10.5281/zenodo.15685613; ref. 29). Exported data are provided in the supplementary files as specified in the “Results” section. The cell packing ratios were determined by taking the total pixel area of all cells that were segmented in an image divided by the total pixel area of the field of view.

### Statistics

Unless stated otherwise, quantitative data were analyzed by the two-tailed unpaired *t* test and are shown as the mean ± SEM. Graphs were made using GraphPad Prism10 (RRID: SCR_002798) software. The number of samples (*n*) refers to biological replicates unless indicated otherwise.

## Results

### Durable alterations of breast cancer cell proliferation in 3D depending on culture paradigm

**Figure 1.**
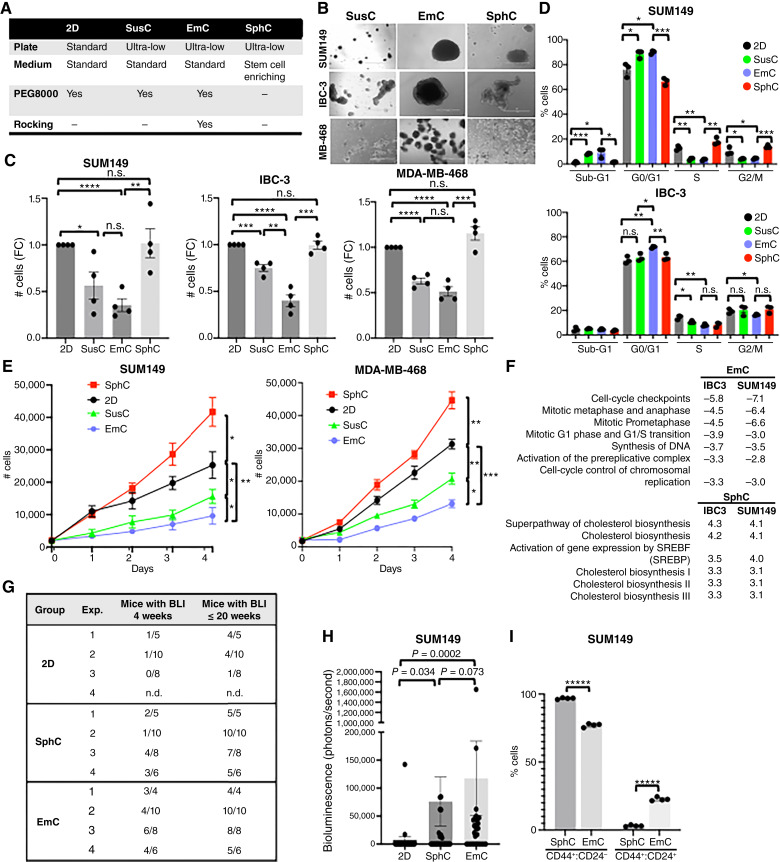
Cells cultured in EmC reduce cell proliferation *ex vivo* but acquire lung colonization efficiency *in vivo*. **A,** Overview of cell culture conditions used in this study (see “Materials and Methods” for further details). **B,** Brightfield images of the of indicated cell lines after 3 days in the specified 3D culture conditions (scale bar, 1 mm). **C,** Viable cell counts after 3 days of culture under the indicated conditions, all starting with equal cell numbers (*n* = 4). **D,** Flow cytometric analysis of DNA content of the cells from SUM149 and IBC-3 cells cultured as in **C** (*n* = 3). **E,** Quantification of viable cells by imaging cytometry replated in 2D (day 0) after 3 days under the indicated culture conditions (*n* = 3). Statistical analysis refers to day 4. **F,***Z*-scores with *P* < 0.05 derived from IPA analysis of common DEGs in EmC and SphC, respectively, compared with all other conditions. **G,** Number of mice across four independent experiments, showing lung BLI after tail vein injection of SUM149 cells that had been cultured as 2D, SphC, and EmC for 3 days before injection by the tail vein. Data are for measurements at week 4 and cumulative numbers until week 20 after injection. *P* values were determined by Fischer exact test as follows: 2D vs. SphC, *P* = 0.045; 2D vs. EmC, *P* = 0.00013; SphC vs. EmC, *P* = 0.065. **H,** Quantification of bioluminescence of all mice at week 4 after injection (*n* = 22–29). *P* values were determined by Wilcoxon test (unpaired, two-sided). **I,** Flow cytometric quantification of SUM149 cells exhibiting CD44^+^:CD24^−^ and CD44^+^:CD24^+^ surface marker phenotypes when cultured in SphC or EmC (*n* = 4). All quantitative data, except (**G** and **H**), are mean ± SEM, *, *P* < 0.05; **, *P* < 0.01; ***, *P* < 0.001; ****, *P* < 0.0001; n.s., not significant.

In this study, we examined four culture conditions (Fig. 1A) using three epithelial breast cancer cell lines (SUM149, IBC-3, and MDA-MB-468), which were chosen after comparing several breast cancer cell lines for their ability to form 3D assemblies in EmC (Supplementary Fig. S1A). In contrast to the original report of this culture method (19), we observed that all cell lines formed “emboli,” with those by basal epithelial cell lines seeming most compact. Apart from conventional 2D culture of these adherent cells on plastic, all other conditions were in ultra-low attachment plates. Because EmC requires addition of PEG8000 to standard, cell line–specific media (19), PEG8000 was also added to 2D and suspension culture (SusC) to control for the effects of media viscosity and molecular crowding. Thus, comparison of 2D and SusC reveals the effect of losing substrate attachment. Comparison of SusC and EmC provides information on the effect of the dynamic nature of EmC, which is the only difference between these two types of culture. The media composition is the only difference between SusC and SphC cultures which were in specific medium that enriches stem cell–like features (30). EmC and SphC differ in both media composition and dynamic versus static conditions. Brightfield imaging of 3-day cultures revealed characteristic structures formed under each 3D condition (Fig. 1B). Cell clusters were smallest in SusC, in which loss of substrate attachment limits aggregation, and largest in EmC, likely reflecting enhanced aggregation and reinforced cell–cell adhesions under fluid dynamic forces. Comparison of the numbers of cells within 3D structures (isolated by brief low-speed centrifugation) suggested that loss of substrate by SusC reduced the cell proliferation rate, whereas the media in SphC rescued proliferation, likely because it is optimized for stemness (Fig. 1C and D). Analysis of cell-cycle stages by DNA content in SUM149 and IBC-3 cells supported this conclusion and indicated that cell death (sub-G1 DNA content) was minimal (Fig. 1D). In addition, staining for the proliferation marker Ki67 was highest in 2D and SphC and reduced but still present in SusC and EmC (Supplementary Fig. S1B). Notably, when cells from 3D culture were dissociated and replated in 2D, these proliferation rate differences persisted for at least 4 days (Fig. 1E). Compared with cells never exposed to 3D culture, cells from SphC proliferated more rapidly, whereas cells from SusC, and especially EmC, showed a durable reduction in their proliferation rates. These results indicate that 3D culture conditions impart lasting cell adaptations that are detectable at the level of proliferation. To address molecular differences, we conducted single-cell mRNA-Seq (scSeq) of SUM149 and IBC-3 cells in 2D, SphC, and EmC. UMAP cluster analysis revealed that each population of SUM149 cells segregated well with only limited overlap of clusters containing cells of two or more culture conditions (Supplementary Fig. S2A and S2B). For IBC-3, cells in 2D and EmC were more similar, whereas those in SphC segregated the most. Next, we identified DEGs for each condition in bulk to all others (Supplementary File S1) and performed pathway analyses (Supplementary File S2). Comparison of the pathways with most significant *z*-scores validated the observed reduction in cell proliferation in EmC whereas cholesterol biosynthesis was upregulated in SphC (Fig. 1F). Increased cholesterol biosynthesis may be consistent with the higher proliferation rate in SphC, although mitosis-related pathways were only upregulated in IBC-3 in SphC (Supplementary File S2). Collectively, these results demonstrate that cancer cells durably and differentially alter their cell proliferation rates in SphC versus EmC.

Mammosphere culture is known to enrich for CSCs in cell lines, increasing the tumor take rate and experimental metastasis rate (31, 32). In contrast, the reduced proliferation rate of cells in EmC could potentially indicate senescence or dormancy. Pilot experiments determining these states were inconclusive, so we next assessed the capacity of SUM149 cells, which express a luciferase reporter, to seed experimental metastases. Cells from 3D cultures were dissociated into single cells and injected by the tail vein into immunocompromised mice; 2D-cultured cells served as controls. Bioluminescence intensity (BLI) was determined on the same day to confirm lung seeding. As anticipated, most injected cells died during the initial phase after injection, and 4 weeks later most mice did not show any BLI (Fig. 1G). In pilot experiments, we had determined that injection of 10^5^ cells was suitable to demonstrate the enhanced colonization by cells from SphC as SphC induces the generation of cancer stem-like and metastasis-initiating cells (32, 33). Accordingly, the frequency of mice with BLI signal was higher in mice which received cells from SphC (34.5%) compared with 2D controls (8.7%). Even more mice (60.7%) injected with cells from EmC exhibited lung colonization, but this trend was not statistically different from SphC. BLI quantification confirmed that EmC increased lung colonization efficiency relative to 2D and was similar to that of SphC cells (Fig. 1H). By week 20, nearly all mice injected developed detectable lung colonies (Fig. 1G), which were confirmed by histologic analysis (Supplementary Fig. S3A). Because SphC promotes lung colonization in part by enriching for CSCs with predominantly CD44^+^/CD24^low/−^ cell surface marker expression (32, 33), we analyzed these markers in cells from SphC and EmC by flow cytometry. This approach revealed that SphC contained more CD44^+^/CD24^−^ cells in comparison with EmC, whereas EmC fostered the emergence of CD44^+^/CD24^+^ CSCs (Fig. 1I; Supplementary Fig. S3B), which may be hybrid epithelial, invasive metastasis-initiating cells (34, 35). Although we cannot exclude the possibility that re-establishment of cell–cell adhesions between cells from EmC cultures enhanced initial survival after injection (33), these results demonstrate that EmC does not diminish cell malignancy. Rather, despite their lower proliferation rate in culture, EmC-derived cells exhibited efficient metastatic competence similar to cells from SphC, which may be in part due to the emergence of epithelial metastasis-initiating cells.

### SUM149 ultrastructural features in EmC bear most resemblance to primary tumors

**Figure 2.**
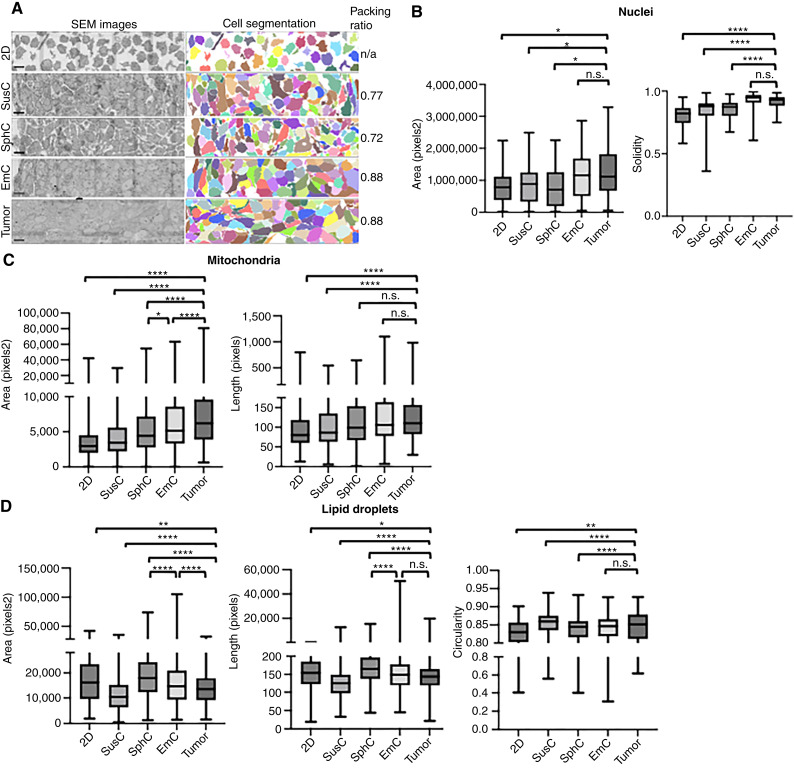
Ultrastructural analysis of SUM149 cell cultures and tumor tissue. **A,** Representative scanning electron microscope (SEM) images and corresponding cell segmentation of SUM149 cells after 3 days in the indicated culture conditions, as well as from a xenograft tumor. Scale bar, 10 μm. **B,** Nuclear metrics of the indicated features for each condition (*n* = 50–68). **C,** Mitochondrial metrics of the indicated features for each condition (*n* = 1,451–1,995). **D,** LD metrics of the indicated features for each condition (*n* = 131–654). *P < 0.01, **, *P* < 0.001; ****, *P* < 0.00001; n.s., not significant.

Given the distinct morphologies of cell assemblies across the three 3D conditions (Fig. 1B), we used volume electron microscopy, specifically array tomography (36), to assess the ultrastructural adaptations of SUM149 cells and their resemblance to *in vivo* tumor tissue. As shown in Fig. 2A, electron microscopy images revealed that the cell packing ratio was highest and most similar in EmC and tumor tissue. Cells from 2D culture were included in this analysis, but packing ratio does not apply to this condition. In addition, mitochondria and nuclei seemed largest, and cell-to-cell connectedness most uninterrupted, in EmC and tumors (Supplementary Fig. S4A and S4B). Artificial Intelligence–based algorithmic quantification of nuclear and mitochondrial morphometrics in one sample per condition further underscored these similarities. Nuclear analyses showed that area and solidity, signifying mostly convex or positive curvature of the membrane, were highest and most similar in tumor and EmC samples (Fig. 2B). Nuclear aspect ratios (length/width) and perimeters were similar across culture conditions and tumor tissue (Supplementary Fig. S4C). The data indicate that nuclear morphology in EmC resembles the more “normalized” phenotype of tumors, though increased nuclear size has also been correlated to neoplastic malignancy (37). Mitochondrial analysis revealed that cells in EmC and tumor tissue harbored the largest mitochondria by area and major axis length, whereas mitochondria in SphC were the least circular with the highest aspect ratios (Fig. 2C; Supplementary Fig. S4D). To extend this analysis, we developed new algorithms to examine LD morphology. Cells in SphC contained the largest LDs with the greatest area and major axis length, whereas LDs in EmC more closely resembled those in tumor tissue, showing similarity in major axis length, circularity, and staining intensity (Fig. 2D; Supplementary Fig. S4E and S4F). Although the determinants of LD staining intensity remain unclear, we speculate that the lipid composition could be a determining factor. In contrast to a prior ultrastructural study of SUM149 emboli (38), we did not observe many microvilli. Full quantifications and statistical analyses are provided in Supplementary Files S3 and S4. Together, these data suggest that SUM149 cells adopt distinct ultrastructural features depending on culture condition and that EmC recapitulates certain aspects of mitochondrial, nuclear, and LD morphology observed in tumor tissue *in vivo*.

### Adaptation of mitochondrial metabolism depends on 3D paradigm

**Figure 3.**
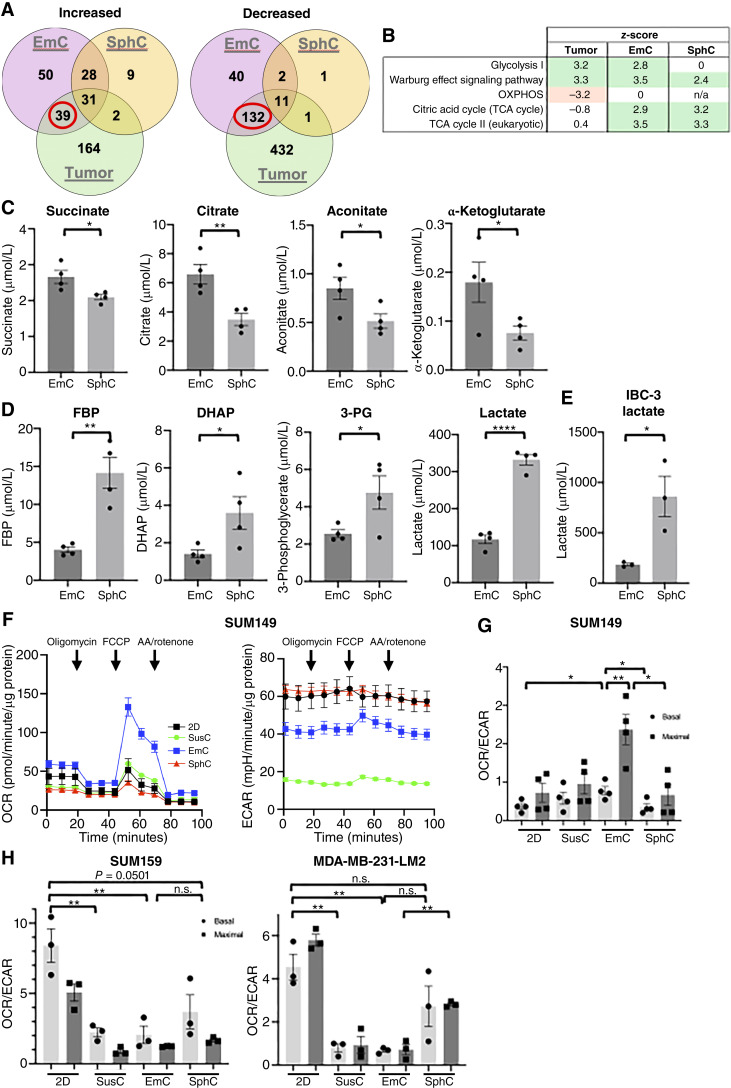
Proteomic and metabolic analysis reveals differential cell adaptations to 3D culture. **A,** Venn diagram showing the numbers of SUM149 proteins differentially under- or over-represented in EmC, SphC, and tumor samples compared with 2D cultures. **B,** Most significant pathway *z*-scores with *P* < 0.05 in common between primary tumors, EmC, and SphC in comparison with 2D. **C,** Fractional enrichment of the indicated intracellular metabolites of SUM149 cells in SphC and EmC (*n* = 4). **D,** Fractional enrichment of the indicated intracellular metabolites of SUM149 cells in SphC and EmC (*n* = 4). **E,** Intracellular lactate levels in IBC-3 cells under the indicated culture conditions (*n* = 3). **F,** OCR and ECAR over time for SUM149 cells derived from the indicated culture conditions. Shown are a representative data normalized to protein, with three technical replicates per time point. **G,** OCR/ECAR ratios of SUM149 cells as in **F**, data are from four independent experiments each with three time points and 3 to 4 technical replicates. **H,** OCR/ECAR ratios of SUM159 and MDA-MB-231-LM2 cells from three independent experiments each with three time points and 3 to 4 technical replicates. Bar graphs show means ± SEM, *, *P* < 0.05; **, *P* < 0.01; ****, *P* < 0.0001; n.s., not significant.

To probe molecular adaptations to different growth conditions, we conducted proteomic analysis of the total levels of proteins in 2D, SphC, and EmC compared with xenograft tumor tissues (Supplementary File S5). Principal component analysis plots of the data from culture conditions (2D, SphC, and EmC) and primary tumors showed well separated clustering of the biological replicates (Supplementary Fig. S5A). Using the 2D proteome as reference, we compared the DEPs between conditions (Fig. 3A; Supplementary Fig. S5B). As expected, the more complex and heterogenous tumor tissue was most divergent from cells cultured in 2D (236 up, 576 down). Cells from SphC showed the least DEPs (70 up, 15 down), whereas cells from EmC were intermediate (148 up, 159 down). Cells from EmC showed the most overlap with tumor tissue (Fig. 3A). Analysis of the proteins exclusively overrepresented in EmC and tumors by ToppGene (39) showed that 35 of 39 were part of the Metabolism Reactome (GSEA ID MM14563), whereas 116 of 132 underrepresented proteins were part of the RNA Metabolism Reactome (GSEA ID M16843), encompassing splicing and translation. Ingenuity Pathway Analysis (Supplementary Fig. S5B; Supplementary File S5) further showed that translational activity was underrepresented in tumors and in EmC, consistent with the reduced proliferation rate in EmC compared with 2D (Fig. 1D). Regarding metabolic pathways, not surprisingly, tumor contained more proteins in glycolysis and less in oxidative phosphorylation (OXPHOS) pathways (Fig. 3B). In contrast, both EmC and SphC showed increases in tricarboxylic acid (TCA) cycle pathway as well as glycolysis and/or Warburg effect. Collectively, the proteomic analysis showed distinct and common adaptations of cells to different 3D culture conditions and suggests that cells in EmC share more similarities with tumor tissue than do cells in SphC.

To further examine metabolic aspects, we determined the levels of central carbon metabolites in SUM149 cells by MS. Four TCA cycle metabolites were enriched in EmC compared with SphC (Fig. 3C). Similar observations were made at the level of pyruvate and glutamate, which feeds into the TCA cycle, and NADH, which is largely produced by the TCA cycle and fuels the electron transfer chain (ETC; Supplementary Fig. S5C). In contrast, four glycolysis pathway intermediates, including lactate, were enriched in SphC in comparison with EmC (Fig. 3D). Other metabolites that were measured were similar between conditions, namely malate, fumarate fructose-6-phosphate, phosphoenolpyruvate, glucose-6-phosphate, and glyceraldehyde-3-phosphate. Elevated levels of lactate in SphC over EmC were also observed in IBC-3 cells (Fig. 3E). Whereas steady-state levels of metabolites alone do not allow conclusions on the activity of metabolic enzymes, these data suggested that TCA cycle activity of cells in EmC may drive OXPHOS whereas cells in SphC may rely on glycolysis. To test this hypothesis directly, we proceeded to functionally examine metabolic activities by Seahorse metabolic flux technology. For this, the 3D cell structures were dissociated and single cells seeded for analysis. The basal and maximal energy metabolism of cells in different culture conditions were assessed by analyzing OCR and ECAR ratios. OCR is a measure of mitochondrial respiration and OXPHOS, and ECAR is a measure of glycolytic activity. Data in Fig. 3F and G showed that SUM149 cells in EmC exhibited the highest basal and maximal OCR/ECAR ratios. IBC-3 and MDA-MB-468 cells demonstrated overall higher metabolic activity. Nevertheless, cells in EmC exhibited the highest OCR/ECAR ratio (Supplementary Fig. S5D and S5E).

To evaluate the generalizability of this observation, we examined two mesenchymal TNBC cell lines, SUM159 and MDA-MB-231-LM2. Because SUM159 could form large “emboli” in EmC (Supplementary Fig. S5F), we asked whether E-cadherin may be upregulated under these conditions, which was, however, not observed (Supplementary Fig. S5G). Analysis of the metabolic profiles showed that the OCR/ECAR ratios were highest in 2D and significantly reduced in SusC and EmC (Fig. 3H). These results show that cell–cell adhesion in 3D alone is not sufficient to enhance OXPHOS in these mesenchymal-like breast cancer cells but rather suggest that the epithelial phenotype may be important for the metabolic shift. Collectively, our data indicate that EmC supports OXPHOS, whereas SphC supports glycolytic metabolism in epithelial breast cancer cells.

### Specific 3D models reveal distinct drug vulnerabilities

**Figure 4.**
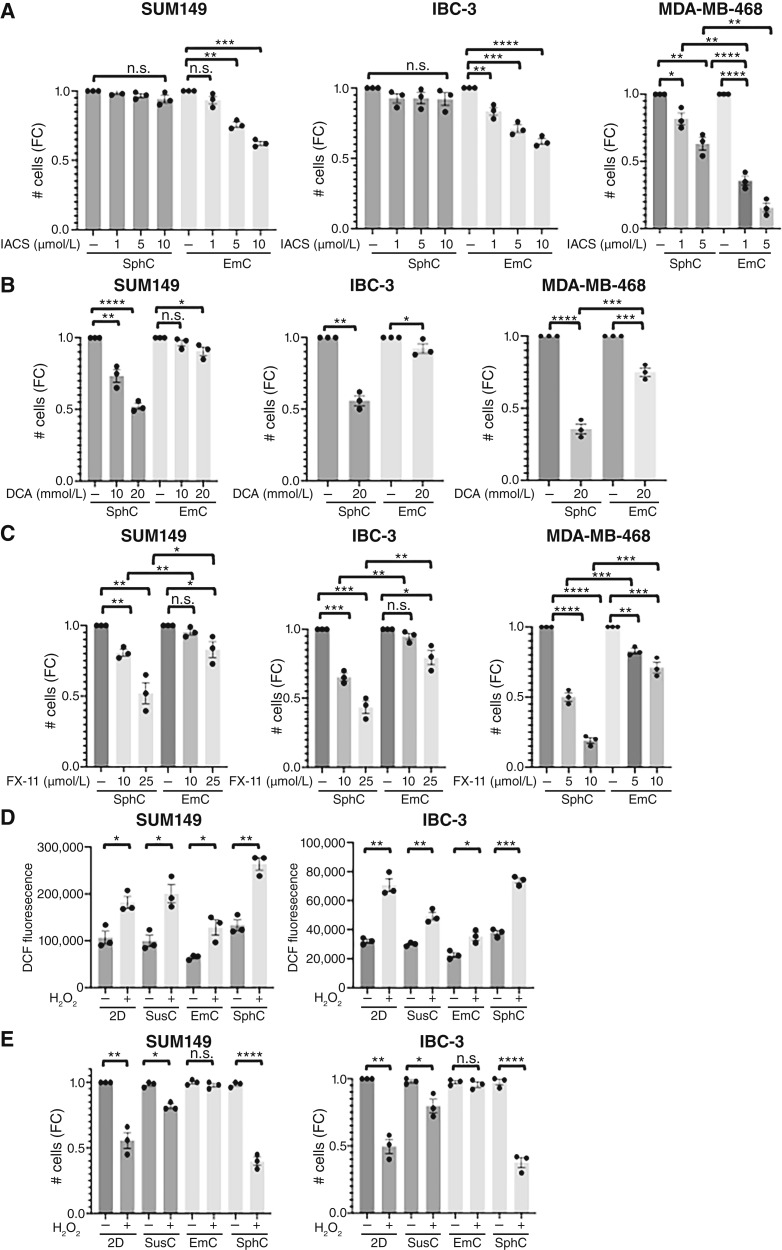
EmC renders cells sensitive to ETC inhibitors and protected from oxidative stress. **A,** FC in the numbers of viable SUM149, IBC-3, and MDA-MB-468 cells in EmC and SphC after 72 hours of treatment with OXPHOS inhibitor IACS-010579, added at the indicated concentrations to the established cultures. DMSO was used as a vehicle control (*n* = 3). **B,** FC in the numbers of viable SUM149, IBC-3, and MDA-MB-468 cells in EmC and SphC after 72 hours of treatment with DCA, added at the indicated concentrations to the established cultures. DMSO was used as a vehicle control (*n* = 3). **C,** FCs in the numbers of viable SUM149, IBC-3, and MDA-MB-468 in EmC and SphC after 72 hours of treatment with FX-11, added at the indicated concentrations to the established cultures. DMSO was used as a vehicle control (*n* = 3). **D,** Quantification of ROS by DCF fluorescence measurements in SUM149 and IBC-3 cells from the indicated culture conditions. H_2_0_2_ (100 μmol/L) was added to the dissociated cells 15 minutes prior to analysis (*n* = 3, mean ± SEM). **E,** FC in the numbers of viable SUM149 and IBC-3 cells as in **C** after 2 hours of exposure to H_2_0_2_. Data are expressed as mean ± SEM, *, *P* < 0.05; **, *P* < 0.01; ***, *P* < 0.001; ****, *P* < 0.0001); n.s., not significant.

Given the differential metabolic pathway activities between cells in EmC versus SphC, we next examined their responses to pathway-specific inhibitors. For OXPHOS, we used IACS-010759, a complex I inhibitor of the ETC (40). In addition, we used DCA, which inhibits pyruvate dehydrogenase kinases and thereby promotes the conversion of pyruvate to acetyl CoA, blocking its conversion to lactate (41). Treatment of established 3D cultures of SUM149 or IBC-3 cells with IACS-010759 for 3 days led to a dose-dependent reduction in cell numbers in EmC (Fig. 4A), accompanied by increased cell death (Supplementary Fig. S6A), whereas SphC cells were completely resistant. Given that IBC-3 cells showed high levels of basal OCR in all culture conditions, the resistance to IACS-010759 in SphC was surprising. MDA-MB-468 cells were not resistant to IACS-010759 in SphC but still less sensitive compared with EmC (Fig. 4A). In contrast, cells in SphC showed significant sensitivity to DCA, whereas cells in EmC were comparatively more resistant (Fig. 4B; Supplementary Fig. S6B). To specifically probe the role of anaerobic glycolysis (Warburg effect), we used the LDH inhibitor FX-11, which was also more effective in SphC compared with EmC in all three cell lines (Fig. 4C). Taken together, these data indicate that cells in SphC rely mostly on glycolysis, which is consistent with previous reports (41, 42). In contrast, cells in EmC are more dependent on OXPHOS for their metabolic sustenance.

Mitochondrial OXPHOS is a source of ROS and may trigger oxidative stress (43). We therefore asked whether cells in EmC may exhibit elevated ROS levels or oxidative stress by staining with the cell-permeable fluorogenic dye H_2_DCFDA (44). Furthermore, we assessed the response to imminent oxidative stress through exposure to exogenous H_2_O_2_. As shown in Fig. 4D, the basal level of H_2_DCFDA fluorescence of SUM149 and IBC-3 cells was in fact lower in EmC-derived cells in comparison with 2D and SphC. As expected, H_2_O_2_ exposure for 15 minutes increased H_2_DCFDA fluorescence in all culture conditions but remained the lowest in EmC. After 2 hours of exogenous H_2_O_2_ exposure, the numbers of viable cells were reduced in 2D and SphC relative to untreated controls but were unchanged in EmC (Fig. 4E). These data indicate that, despite their reliance on OXPHOS, EmC cells are most protected from oxidative stress.

### EmC promotes features associated with lung metastasis

**Figure 5.**
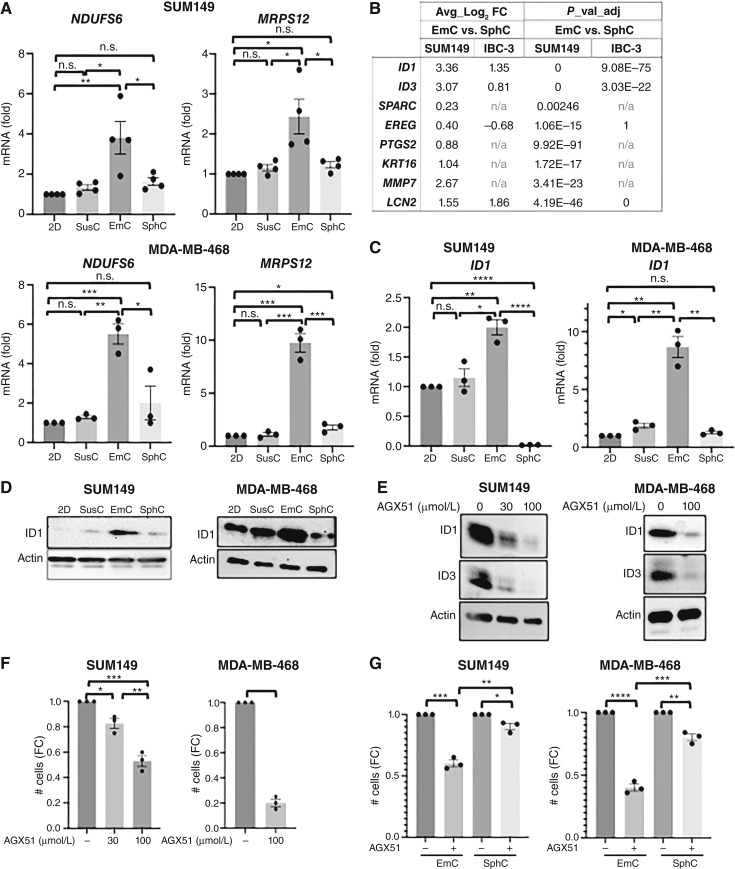
EmC leads to induction of genes related to lung metastasis. **A,** qRT-PCR of mRNA levels for indicated genes in SUM149 and MDA-MB-468 cells under the indicated culture conditions (*n* = 3). **B,** Differential expression of the indicated genes derived from the scRNA-seq data comparing EmC vs. SphC in SUM149 and IBC-3 cells as indicated. **C,** qRT-PCR (top, *n* = 3) of *ID1* expression in SUM149 and MDA-MB-468 cultures as indicated. **D,** Western blot analysis of ID1 expression of cells as in **C**. Actin served as a loading control. **E,** Western blot analysis of ID1 and ID3 proteins in established SUM149 EmC cells that were treated with AGX51 at the indicated concentrations for 3 days. Actin served as a loading control. **F,** FC in viable SUM149 and MDA-MB-468 cells in established EmC following AGX51 treatment at the indicated concentrations for 3 days (*n* = 3). **G,** FC in viable SUM149 and MDA-MB-468 cells in established EmC and SphC following AGX51 treatment (100 μmol/L) for 3 days (*n* = 3). Quantitative data are shown as mean ± SEM, *, *P* < 0.05; **, *P* < 0.01; ***, *P* < 0.001; ****, *P* < 0.0001; n.s., not significant.

Metabolic plasticity is a hallmark of cancer and the metastatic process (45). In epithelial breast cancer, oxidative mitochondrial metabolism and fatty acid oxidation have been associated with metastatic capacity and especially to the lung (46, 47), which is among the most common sites of breast cancer metastasis (48). In EmC, we observed induction of two OXPHOS-related genes, *NDUFS6* and *MRPS12* (Fig. 5A), that are overexpressed in aggressive hormone receptor–negative breast cancer associated with metastasis and poor prognosis (49). Furthermore, analysis of the scRNA-Seq data showed that other genes associated with lung metastases were also enriched by EmC in SUM149 and/or IBC-3 cells: *ID1*, *ID3*, *SPARC*, *EREG*, *PTGS2* (50–52), *KRT16* (53, 54), *MMP7* (55), and *LCN2* (Fig. 5B; ref. 56). Validation experiments confirmed the upregulation of ID1 at both the mRNA and protein levels in SUM149 and MDA-MB-468 cells in EmC (Fig. 5C and D). ID1, an inhibitor of E-box binding transcription factors, is an important regulator of the cell cycle, self-renewal, and tumorigenesis (57–59). Consequently, treatment of cells in EmC with AGX51, which targets ID proteins for degradation (60), reduced ID1 and ID3 protein levels (Fig. 5E) and decreased viable cell numbers in a dose-dependent manner (Fig. 5F; Supplementary Fig. S6C). Consistent with the lower levels of ID1 expression and OXPHOS in SphC, these cells were less sensitive to AGX51 treatment (Fig. 5G). Despite the differences in ID1 levels between *ex vivo* cultures (Fig. 5D), each gave rise to experimental lung metastases that expressed ID1 *in vivo* (Supplementary Fig. S6D). This result agrees with the reported role of ID1 in lung metastasis (51) and suggests that cells from SphC upregulate ID1 *in vivo*, whereas cells in EmC can model this aspect of lung metastases *ex vivo*. Collectively, these results indicate the relevance for ID1/3 upregulation in EmC and suggest their potential role in contributing to the lung-colonizing capacity of EmC-derived SUM149 cells (Fig. 1G and H).

## Discussion

The primary objective of this study was to comprehensively characterize breast cancer cells grown in EmC and compare the findings with more traditional cell culture models such as 2D culture and mammospheres (SphC). Our results indicate three distinct features of EmC as an *ex vivo* model system for epithelial breast cancer: (i) reduced cell proliferation, (ii) an oxidative mitochondrial metabolic profile (OXPHOS), and (iii) ultrastructural and architectural features reminiscent of tumor tissue *in vivo*. These characteristics are crucial because the high proliferation rates in standard *in vitro* systems often make cells artificially sensitive to pharmacologic interventions, leading to false positive results in drug screening and ultimately poor clinical translation (4). In contrast, 3D models, including EmC (7), typically exhibit resistance to drug treatments, making them more appropriate for evaluating therapeutic responses (61). Furthermore, our data suggest that SphC versus EmC may foster the generation of different subtypes of CSCs. These paradigms are, therefore, complementary as model systems to capture the breadth and regulation of metastatic breast cancer cell phenotypes.

Notably, we observed that the fluid dynamics inherent to the EmC system promote tissue-like cell–cell contacts and ultrastructural changes. Whereas previous research has explored the impact of fluid shear stress and tissue stiffness on cancer progression (62, 63), we do not claim that the specific mechanical forces at play in EmC directly model a particular *in vivo* scenario. However, tissues are exposed to pulsatile fluid flow in blood and lymph vessels, which are likely providing mechanical cues. For lung cancer, specifically, the lung environment provides significant mechanical forces that shape tumor development and progression (64). Consistent with this, our transcriptomic analysis revealed upregulation of several genes in EmC that associated with breast cancer lung metastasis. Among these, keratin 16 (*KRT16*), a regulator of mitochondrial dynamics that is upregulated in lung metastases (53, 65), stands out as circulating tumor cells (CTC) expressing *KRT16* are also associated with shorter relapse-free survival (54).

Interestingly, despite robust mitochondrial oxidative activity, EmC cells maintained low levels of ROS, consistent with previous findings that efficient, active TCA cycling is associated with reduced ROS generation (66–68). This observation also aligns with reports that an epithelial phenotype is linked with protection from oxidative stress, potentially mediated through ID1 and KRT16 (65, 69). KRT16 was shown to regulate mitochondrial structure and activity and prevent oxidative stress (65). ID proteins, too, support OXPHOS while protecting against oxidative stress by promoting an adaptive antioxidant–mitochondrial response (69). Therefore, the increased expression of ID1 and KRT16 in EmC may in part contribute to antioxidant mechanisms and explain the lower ROS levels despite increased oxidative mitochondrial metabolism. The mechanosensitive transcription factor YAP has been shown to induce transcription of ID1 (70), which is implicated in lung metastasis initiation (58, 60, 71, 72). Thus, we speculate that YAP-mediated expression of ID proteins may be at least in part responsible for promoting OXPHOS and lung colonization (53, 73).

At the level of energy metabolism, our analyses demonstrate that SphC cells predominately rely on aerobic glycolysis, whereas EmC cells exhibit dependence on mitochondrial OXPHOS. Although glycolytically derived pyruvate canonically feeds the TCA cycle and OXPHOS unless diverted to lactate via the Warburg effect (41, 74), recent studies suggest that lactate itself can serve as a fuel for mitochondrial metabolism (75, 76). However, only EmC cells but not SphC cells were sensitive to ETC inhibition, underscoring their reliance on mitochondrial oxidative metabolism. These observations are especially relevant in the context of metastasis, in which metabolic reprogramming enables tumor cells to adapt and thrive within new microenvironments (77, 78). Studies have shown that OXPHOS is induced in epithelial breast cancer cells, specifically CTCs and metastasis-initiating cells (46, 79–81). Elevated OXPHOS gene expression is correlated with reduced patient survival, resistance to chemotherapy, and is a metabolic vulnerability in therapy-resistant triple-negative breast cancers (82–85). Consistent with this, metastatic breast cancer cells in the lung amplify OXPHOS metabolism (86), possibly due to the oxygen-rich environment (87), and inhibition of OXPHOS specifically impedes tumor cell seeding of the lung—even when the primary tumor itself is more glycolytic (53).

Combined with our findings that EmC-derived cells exhibit efficient colonization of mouse lungs and the prevalence of OXPHOS metabolism, the above reports suggest that EmC may serve as a valuable *ex vivo* paradigm to investigate pathways relevant to lung metastasis. Although most of our analyses were performed using two IBC cell lines, some findings were validated in another epithelial non-IBC TNBC line. In contrast, two mesenchymal TNBC cell lines did not exhibit EmC-promoted OXPHOS. Additionally, given that our prior work identified a pathway through EmC that was also observed in a non-IBC basal–epithelial TNBC PDX model, i.e., COX-2 through AKT/GSK3β to E-cadherin stabilization (7), we postulate that the EmC system may be broadly applicable at least to basal epithelial breast cancers. Indeed, E-cadherin itself may be a potential mediator of OXPHOS (88, 89). Whereas applicability to other cancer types warrants further investigation, the EmC paradigm may be particularly suitable to address the mechanistic relationship of the epithelial phenotype to oxidative metabolism. Identifying *ex vivo* culture models that faithfully recapitulate distinct *in vivo* tumor phenotypes, especially of difficult to study lung metastases, is critical for dissecting the mechanisms that empower metastatic breast cancer cells and for uncovering new therapeutic targets. Our findings establish EmC as a resource-efficient *ex vivo* model that captures the ultrastructural, metabolic, and functional hallmarks of metastatic basal epithelial breast cancer, providing a valuable tool for probing the biology and therapeutic sensitivities of this and potentially other epithelial tumor types.

## Supplementary Material

Supplementary File 1 scSeq_DEGs_vs_all_others

Supplementary File 2 scSeq_pathway_analysis

Supplementary File 3 Morphometric data of electron microscopy images

Supplementary File 4 Morphometry Statistics

Supplementary File 5 Proteomics_data

Supplementary Figure S1 Emboli formation by different breast cancer cell lines

Supplementary Figure S2 Single cell mRNA sequencing data of cells from 2D, SphC, and EmC

Supplementary Figure S3 SUM149 experimental metastases and CD44/CD24 surface markers

Supplementary Figure S4 Ultrastructural analysis of SUM149 mitochondria, nuclei and lipid droplets

Supplementary Figure S5 Proteomic and metabolic analysis

Supplementary Figure S6 Treatment of cells in 3D with metabolic pathway inhibitors

## Data Availability

The single-cell mRNA-seq data generated in this study are publicly available in GEO at GSE309277. Analyzed data are provided as Supplementary Data Files S1 and S2. Proteomic data have been deposited at MassIVE (https://massive.ucsd.edu/; RRID: SCR_013665) with accession number MSV000099222. Analyzed data are provided in Supplementary File S5. The data generated in this study are available upon request from the corresponding authors.
