# Supplementary material for: 3D “Emboli” Culture Models Epithelial Breast Cancer Cell Oxidative Mitochondrial Metabolism with Relevance for Lung Metastasis
Source: Cancer Res Commun. 2026 Mar 19;6(3):600–15. doi: 10.1158/2767-9764.CRC-25-0587 (PMC13012061; doi:10.1158/2767-9764.CRC-25-0587)
Supplement: Supplementary Figure S1 — Emboli formation by different breast cancer cell lines [file crc-25-0587_supplementary_figure_s1_suppsf1.pdf]

Supplementary Figure S1

S1A

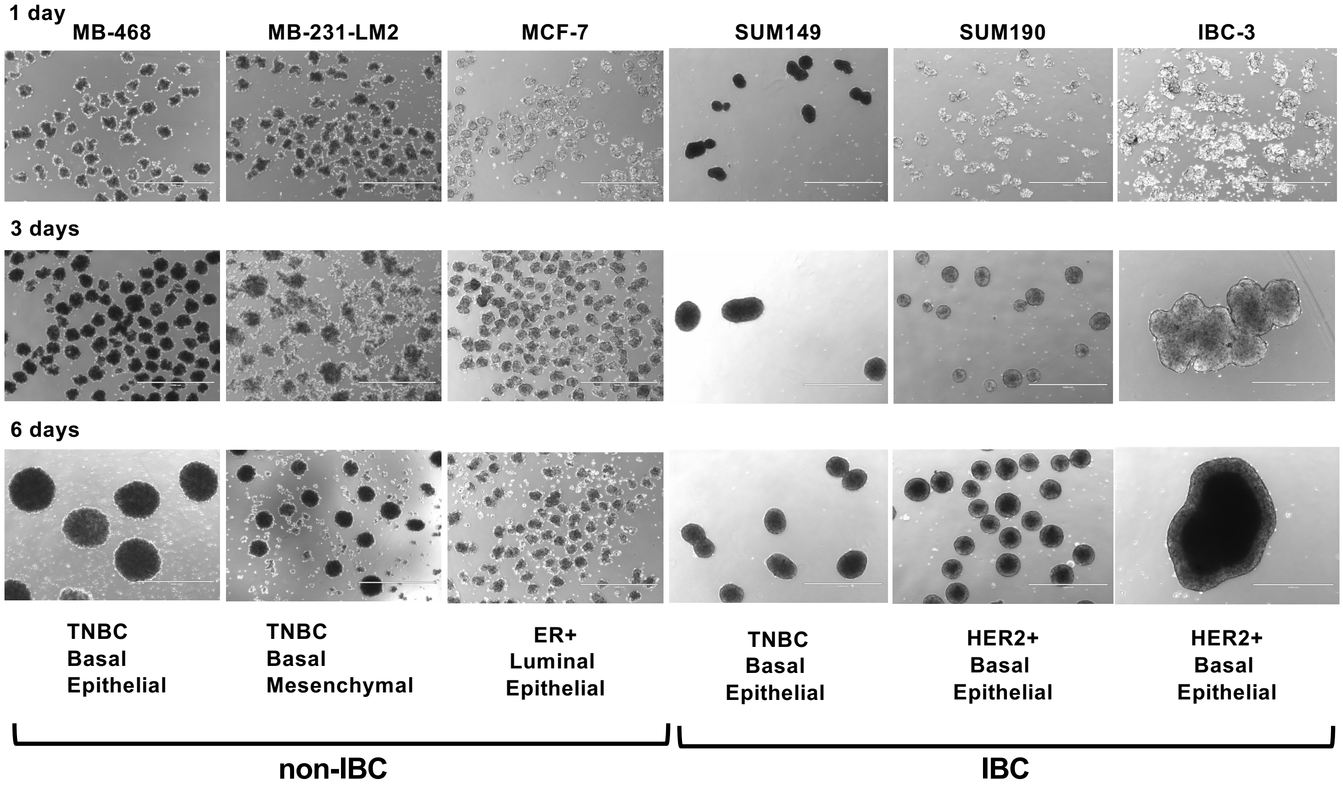

S1B

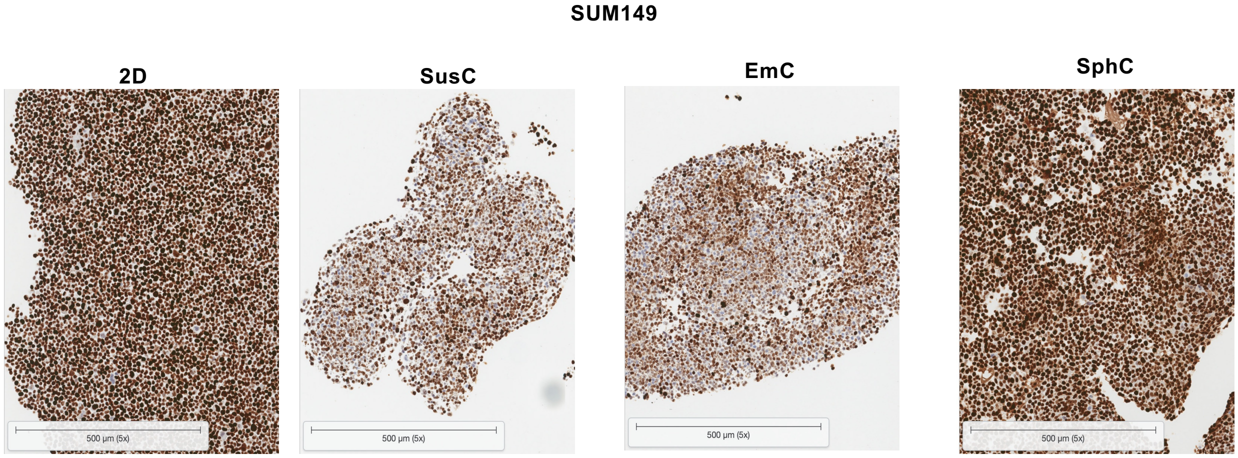

Supplementary Figure S1. Emboli formation by different breast cancer cell lines. A. Light microscopy images of the indicated IBC and non-IBC cells lines after 1, 3, and 6 days in EmC (scale bar=1 mm). **B,** Ki67 Immunochemistry of cell pellets prepared from indicated SUM149 cultures (scale bar = 500 µm).
