# Supplementary material for: 3D “Emboli” Culture Models Epithelial Breast Cancer Cell Oxidative Mitochondrial Metabolism with Relevance for Lung Metastasis
Source: Cancer Res Commun. 2026 Mar 19;6(3):600–15. doi: 10.1158/2767-9764.CRC-25-0587 (PMC13012061; doi:10.1158/2767-9764.CRC-25-0587)
Supplement: Supplementary Figure S2 — Single cell mRNA sequencing data of cells from 2D, SphC, and EmC [file crc-25-0587_supplementary_figure_s2_suppsf2.pdf]

Supplementary Figure S2

S2A **SUM149**

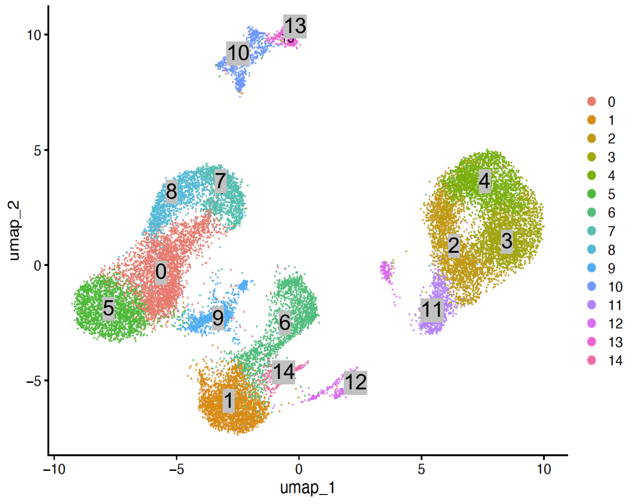

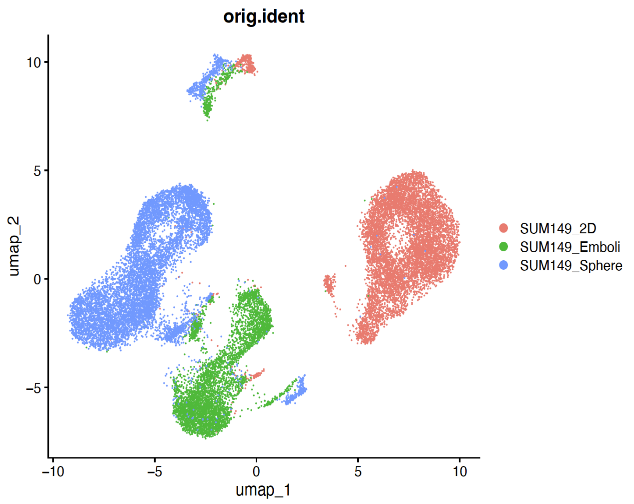

group	0	1	2	3	4	5	6	7	8	9	10	11	12	13	14
SUM149_2D	7	4	2408	2309	2253	2	18	4	2	33	15	725	153	277	64
SUM149_Emboli	6	2486	0	0	2	8	1818	3	3	259	294	1	103	17	164
SUM149_Sphere	3022	36	1	2	1	2225	33	1269	1269	468	444	3	199	7	13

S2B **IBC-3**

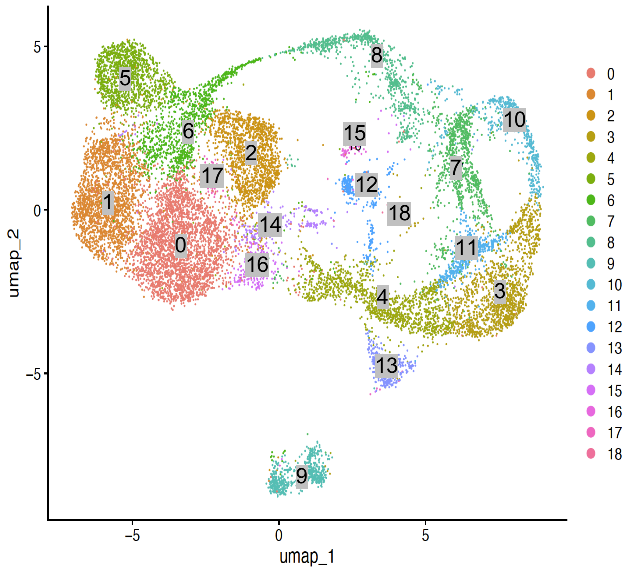

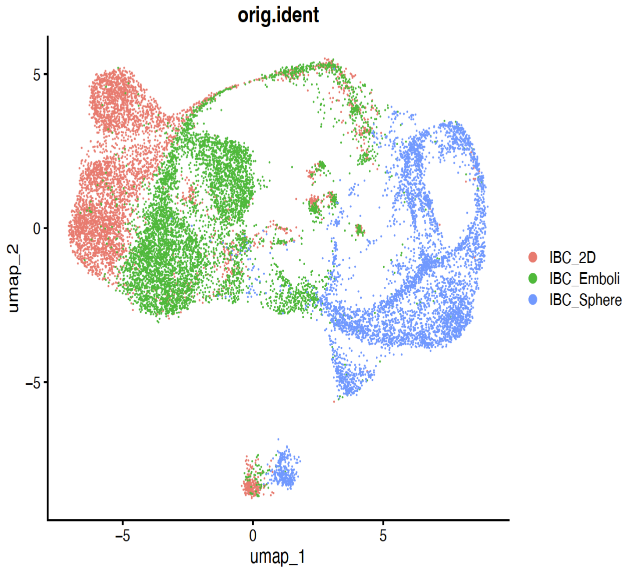

group	0	1	2	3	4	5	6	7	8	9	10	11	12	13	14	15	16	17	18
IBC_2D	115	2135	48	3	1	1251	563	9	294	209	7	0	113	0	104	64	51	50	33
IBC_Emboli	2745	36	1507	8	376	8	525	24	486	57	14	2	126	12	137	188	47	35	35
IBC_Sphere	5	0	1	1431	883	0	2	887	21	300	501	519	108	303	24	6	0	1	0

Supplementary Figure S2. Single cell mRNA sequencing of cells from 2D, SphC, and EmC.
UMAP clusters of scRNA-Seq data derived from (A) SUM149 and (B) IBC-3 cells after 3 days in the indicated culture conditions, along with tables showing the number of cells per cluster and condition. Data represent the combination of two biological replicates each.
