# Supplementary material for: 3D “Emboli” Culture Models Epithelial Breast Cancer Cell Oxidative Mitochondrial Metabolism with Relevance for Lung Metastasis
Source: Cancer Res Commun. 2026 Mar 19;6(3):600–15. doi: 10.1158/2767-9764.CRC-25-0587 (PMC13012061; doi:10.1158/2767-9764.CRC-25-0587)
Supplement: Supplementary Figure S3 — SUM149 experimental metastases and CD44/CD24 surface markers [file crc-25-0587_supplementary_figure_s3_suppsf3.pdf]

Supplementary Figure S3

S3A

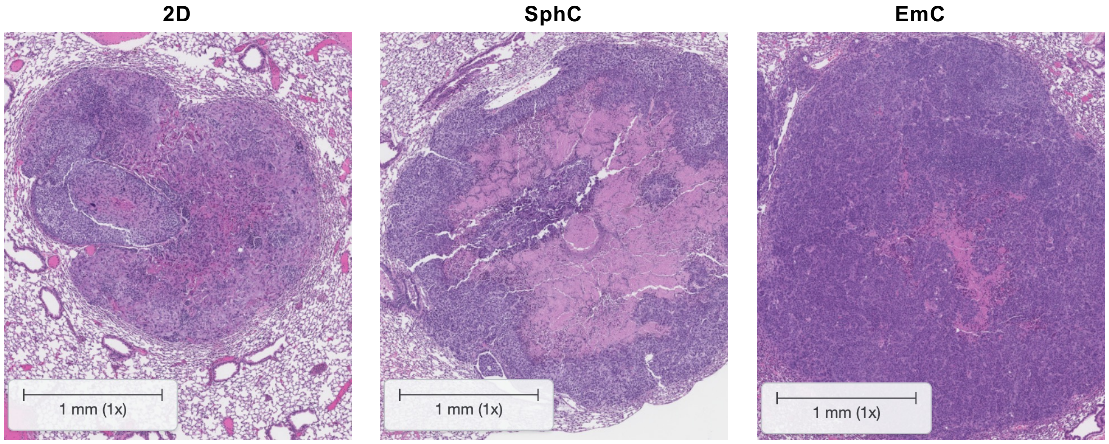

S3B

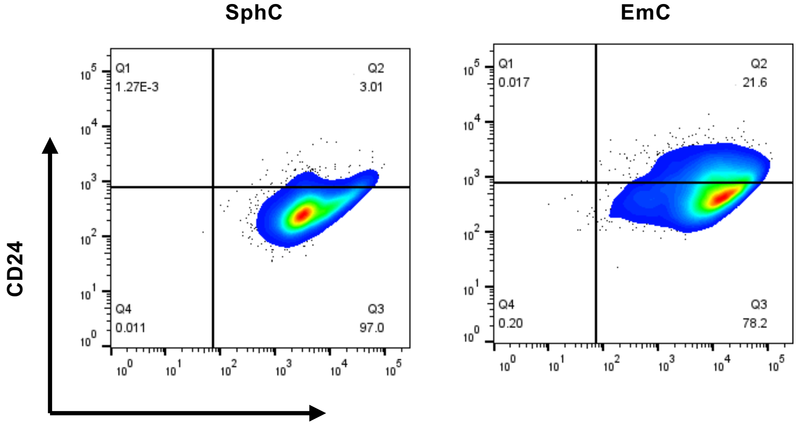

Supplementary Figure S3. SUM149 experimental metastases and C44/CD24 surface markers.

A, Examples of images of H&E staining to validate SUM149 experimental lung metastases generated by cells from the indicated culture conditions (scale bar = 1 mm). **B,** Representative scatter-plots of flow-cytometric analysis of CD44 and CD24 surface marker expression by SUM149 cells after 3 days of SphC or EmC.
