# Supplementary material for: 3D “Emboli” Culture Models Epithelial Breast Cancer Cell Oxidative Mitochondrial Metabolism with Relevance for Lung Metastasis
Source: Cancer Res Commun. 2026 Mar 19;6(3):600–15. doi: 10.1158/2767-9764.CRC-25-0587 (PMC13012061; doi:10.1158/2767-9764.CRC-25-0587)
Supplement: Supplementary Figure S4 — Ultrastructural analysis of SUM149 mitochondria, nuclei and lipid droplets [file crc-25-0587_supplementary_figure_s4_suppsf4.pdf]

Supplementary Figure S4

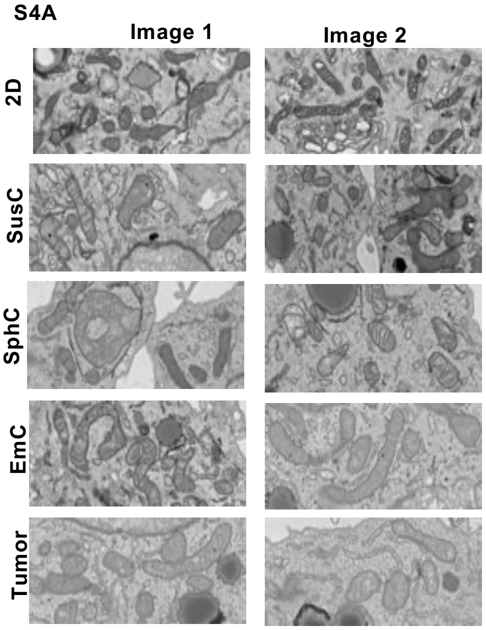

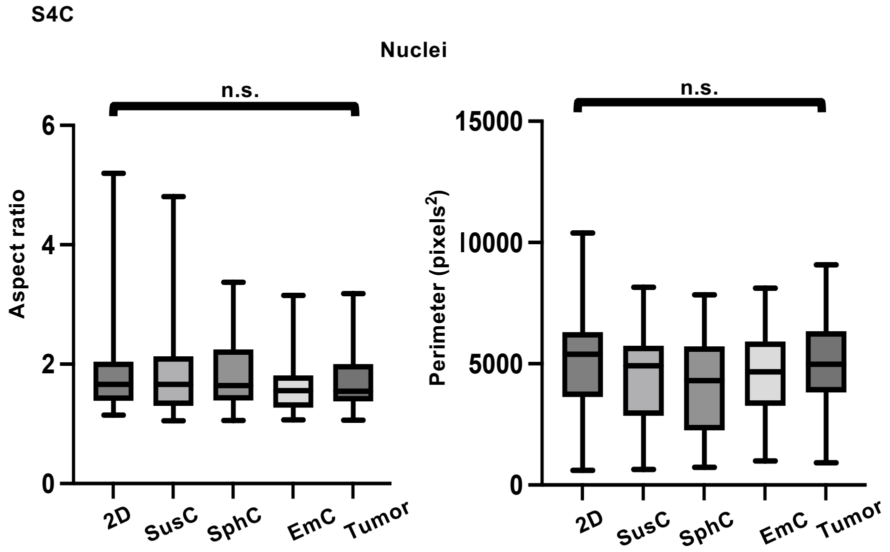

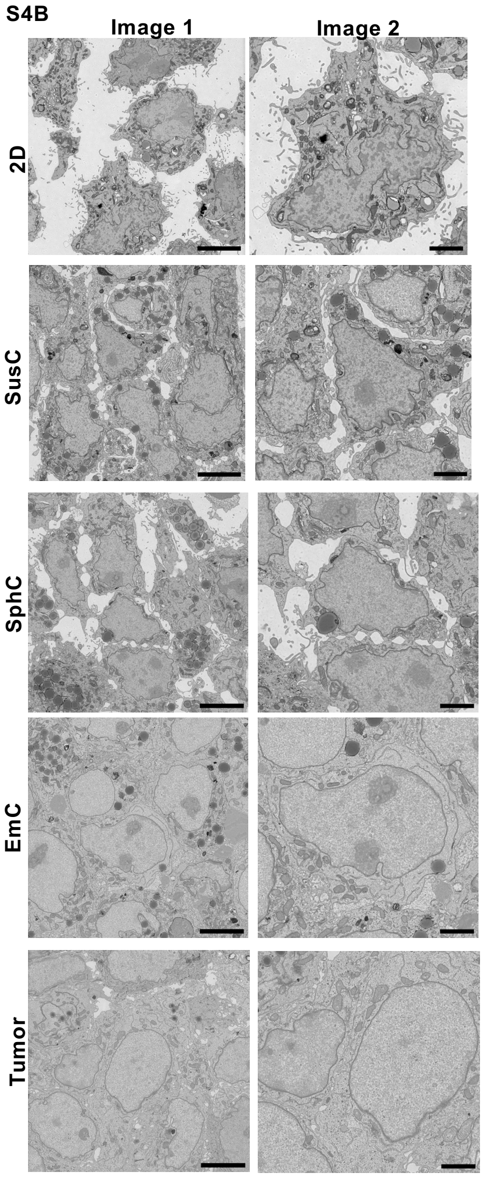

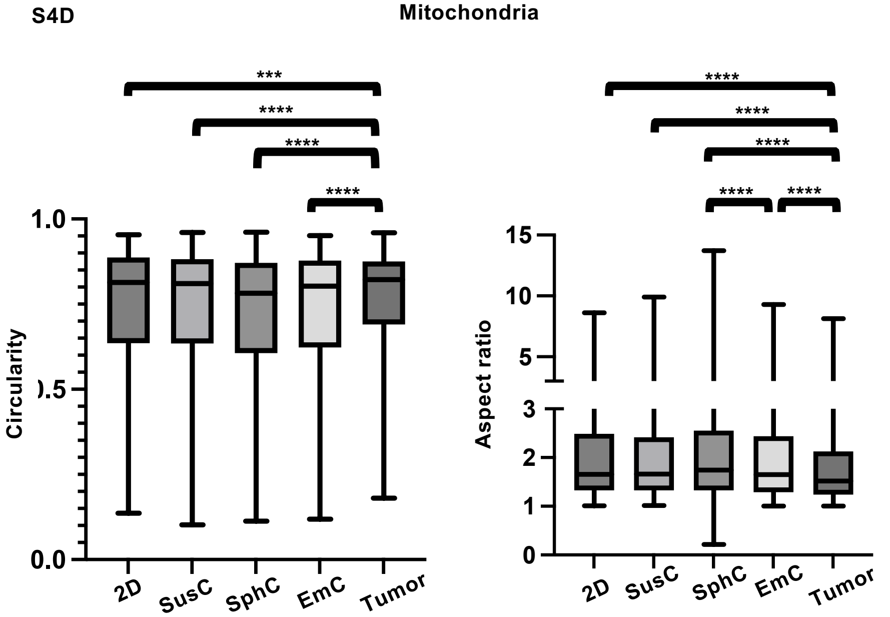

Supplementary Figure S4 continued

S4E

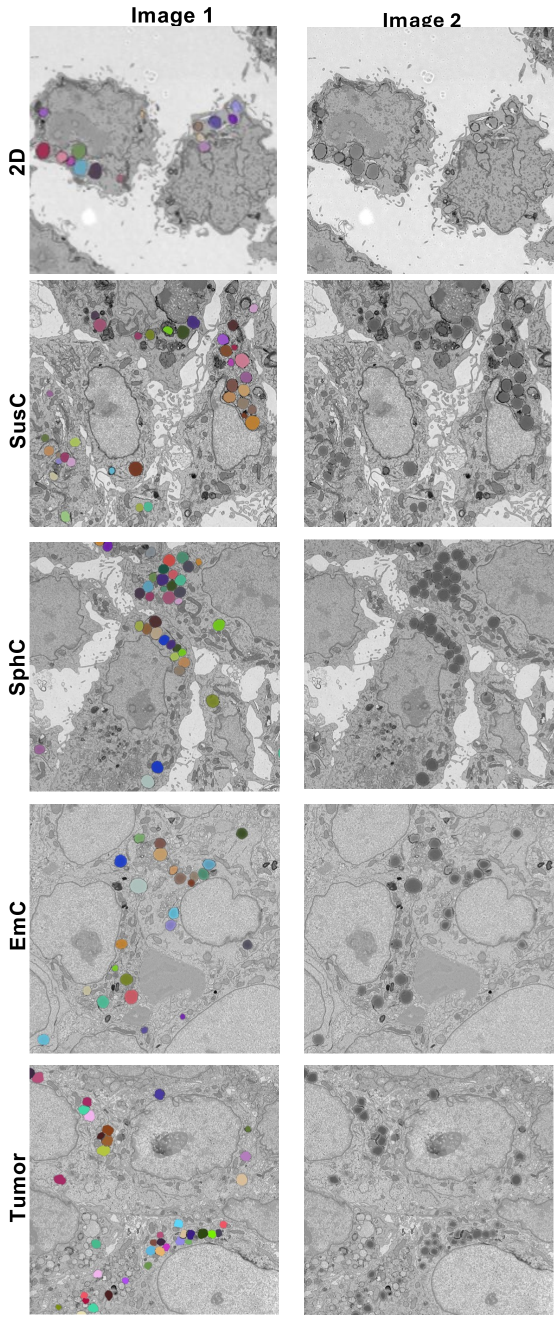

S4F

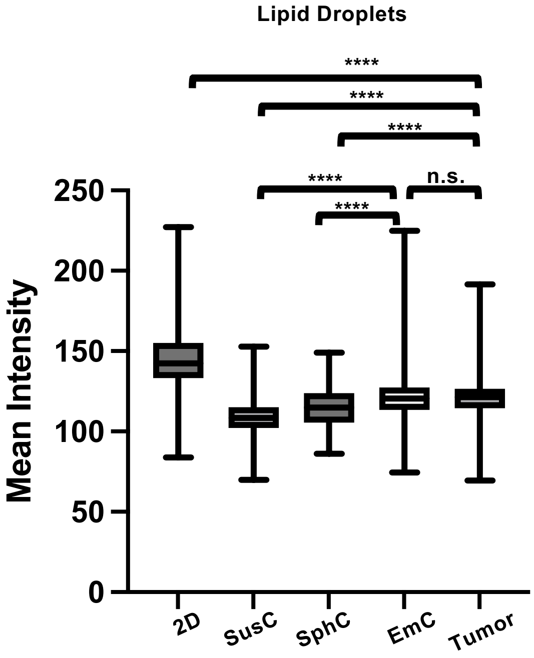

Supplementary Figure S4. Ultrastructural analysis of SUM149 mitochondria, nuclei and lipid droplets. A, Scanning electron microscope images showing mitochondria under indicated conditions. Images taken in FIJI at 25% zoom with 5 µm x 2 µm box. Pixel size = 5 nm. **B,** Scanning electron microscope images of nuclei as in A. Scale bar = 2 µm. **C,** Morphometric data of nuclei in cells cultured as indicated (n = 50-68, mean). **D,** Morphometric data of mitochondria in cells cultured as indicated (n = 1451-1995). **E,** Scanning electron microscope images of lipid droplets LDs (as identified in the image on the left) in cells cultured as indicated. Pixel size=5 nm. **F,** Morphometric data of lipid droplets in cells cultured as indicated (n = 131-654). ***P<0.0001, ****P<0.00001, n.s. not significant.
