# Supplementary material for: 3D “Emboli” Culture Models Epithelial Breast Cancer Cell Oxidative Mitochondrial Metabolism with Relevance for Lung Metastasis
Source: Cancer Res Commun. 2026 Mar 19;6(3):600–15. doi: 10.1158/2767-9764.CRC-25-0587 (PMC13012061; doi:10.1158/2767-9764.CRC-25-0587)
Supplement: Supplementary Figure S5 — Proteomic and metabolic analysis [file crc-25-0587_supplementary_figure_s5_supps5f.pdf]

Supplementary Figure S5

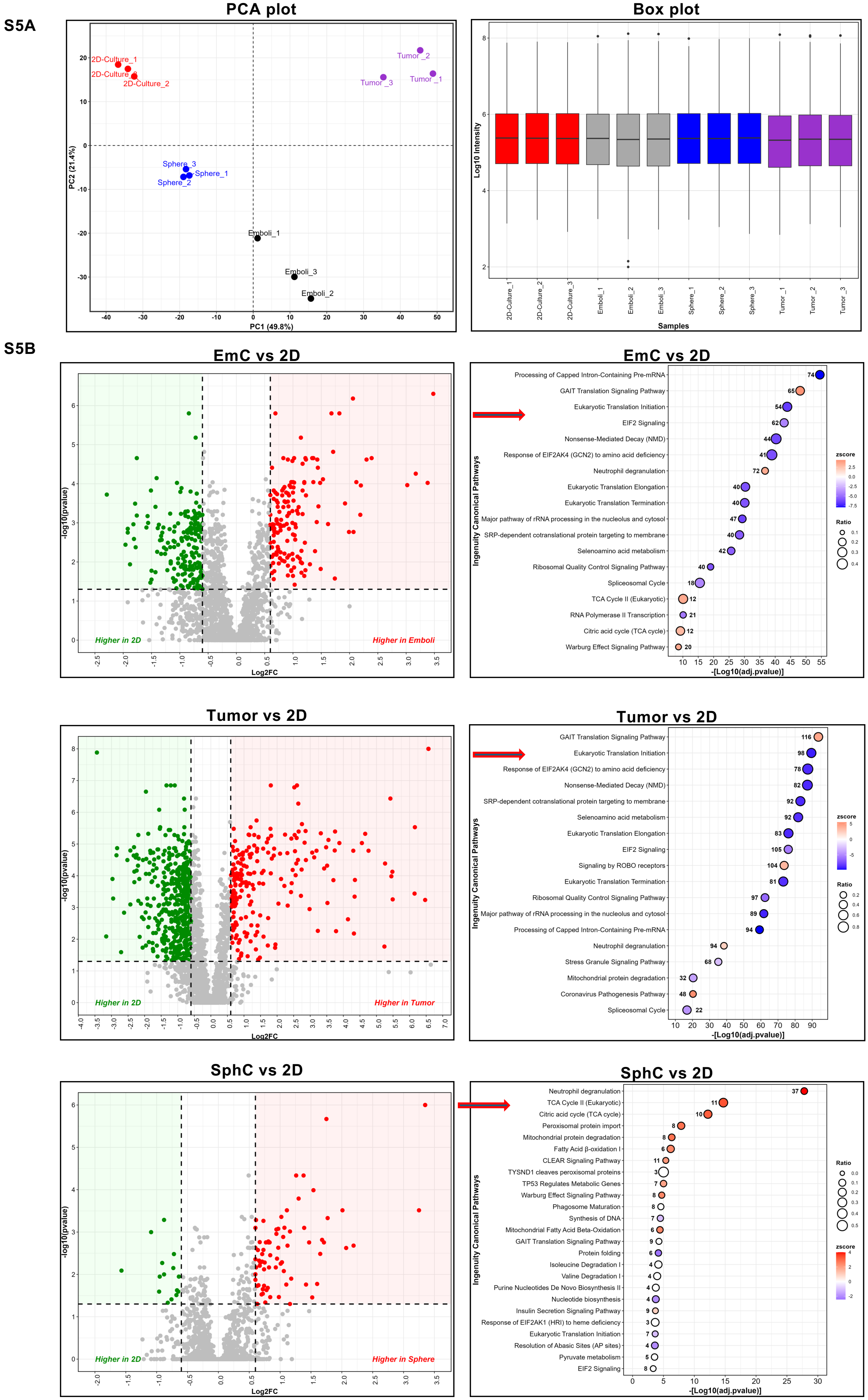

Supplementary Figure S5 continued

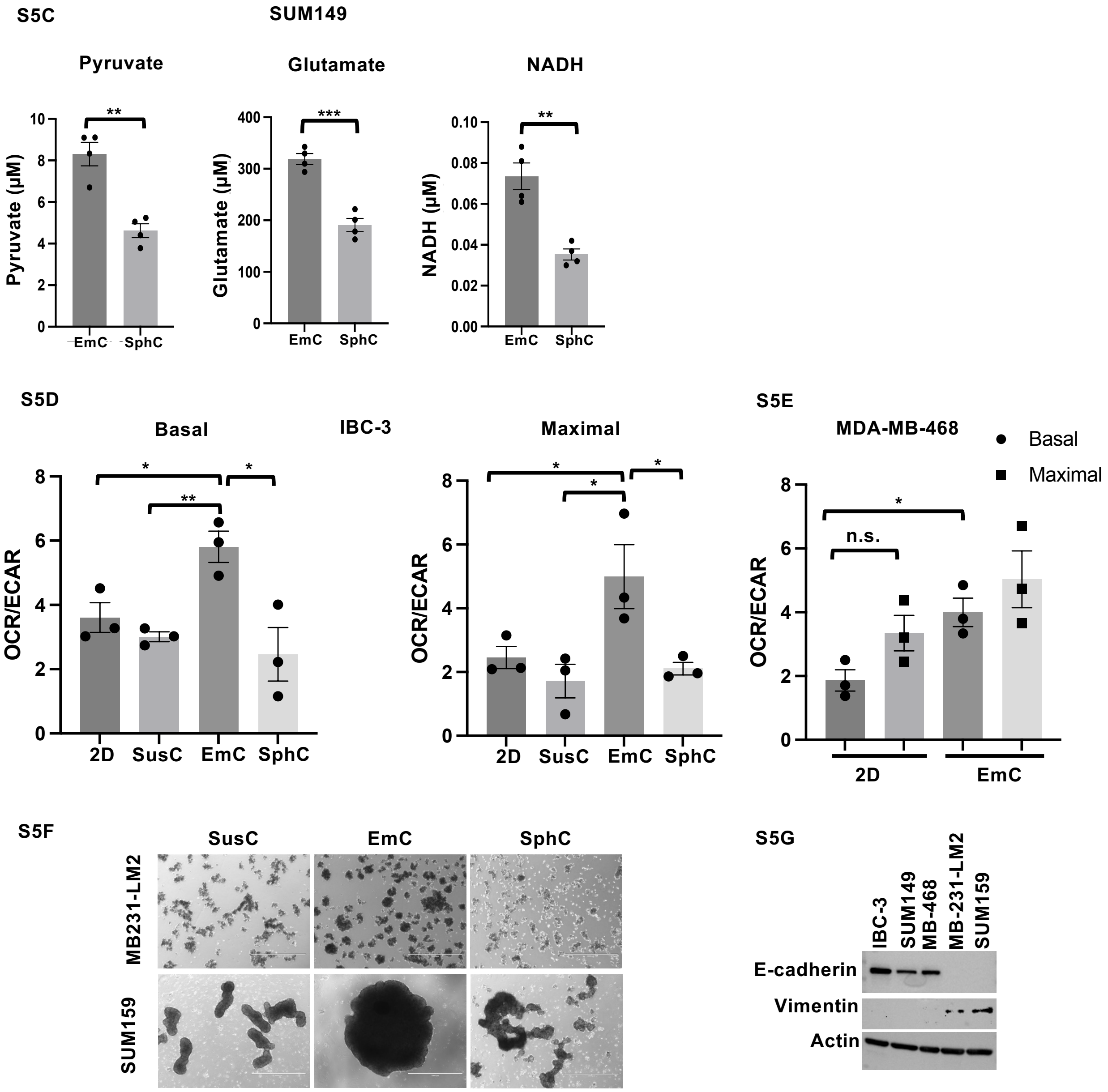

Supplementary Figure S5. Proteomic and metabolic analysis reveals differential cell adaptations to 3D culture. A, PCA and box plots of proteomic data (Supplemental File 5) from SUM149 cells in different culture conditions and xenograft tumor tissue (n = 3). **B,** Volcano plots (left) and Ingenuity Pathway Analyses (right) of protein expression data as in panels A. Arrows point to pathways discussed in the Results. **C,** Intracellular pyruvate, glutamate and NADH concentrations in SUM149 cells cultured for 3 days in SphC and EmC (n = 4). **D,** OCR/ECAR ratio (basal and maximal) in IBC-3 cells after 3 days under the indicated culture conditions. Data are from 3 different experiments with 3 time points and 3-4 technical replicates each. **E,** OCR/ECAR ratio (basal and maximal) in MDA-MB-468 cells cultured as 2D and EmC. Data are from 3 different experiments each with 3 time points and 3-4 technical replicates. **F,** Brightfield images of MDA-MB-231-LM2 and SUM159 cells after three days in the specified 3D culture conditions (scale bar =1 mm). **G**, Western analysis of E-cadherin and vimentin expression in cells cultured for three days in EmC. Actin served as loading control. Data in panels D-E are mean ± SEM,**P*<0.05, ***P*<0.01, ****P*<0.001, n.s., not significant.
