# Supplementary material for: 3D “Emboli” Culture Models Epithelial Breast Cancer Cell Oxidative Mitochondrial Metabolism with Relevance for Lung Metastasis
Source: Cancer Res Commun. 2026 Mar 19;6(3):600–15. doi: 10.1158/2767-9764.CRC-25-0587 (PMC13012061; doi:10.1158/2767-9764.CRC-25-0587)
Supplement: Supplementary Figure S6 — Treatment of cells in 3D with metabolic pathway inhibitors [file crc-25-0587_supplementary_figure_s6_suppsf6.pdf]

Supplementary Figure S6

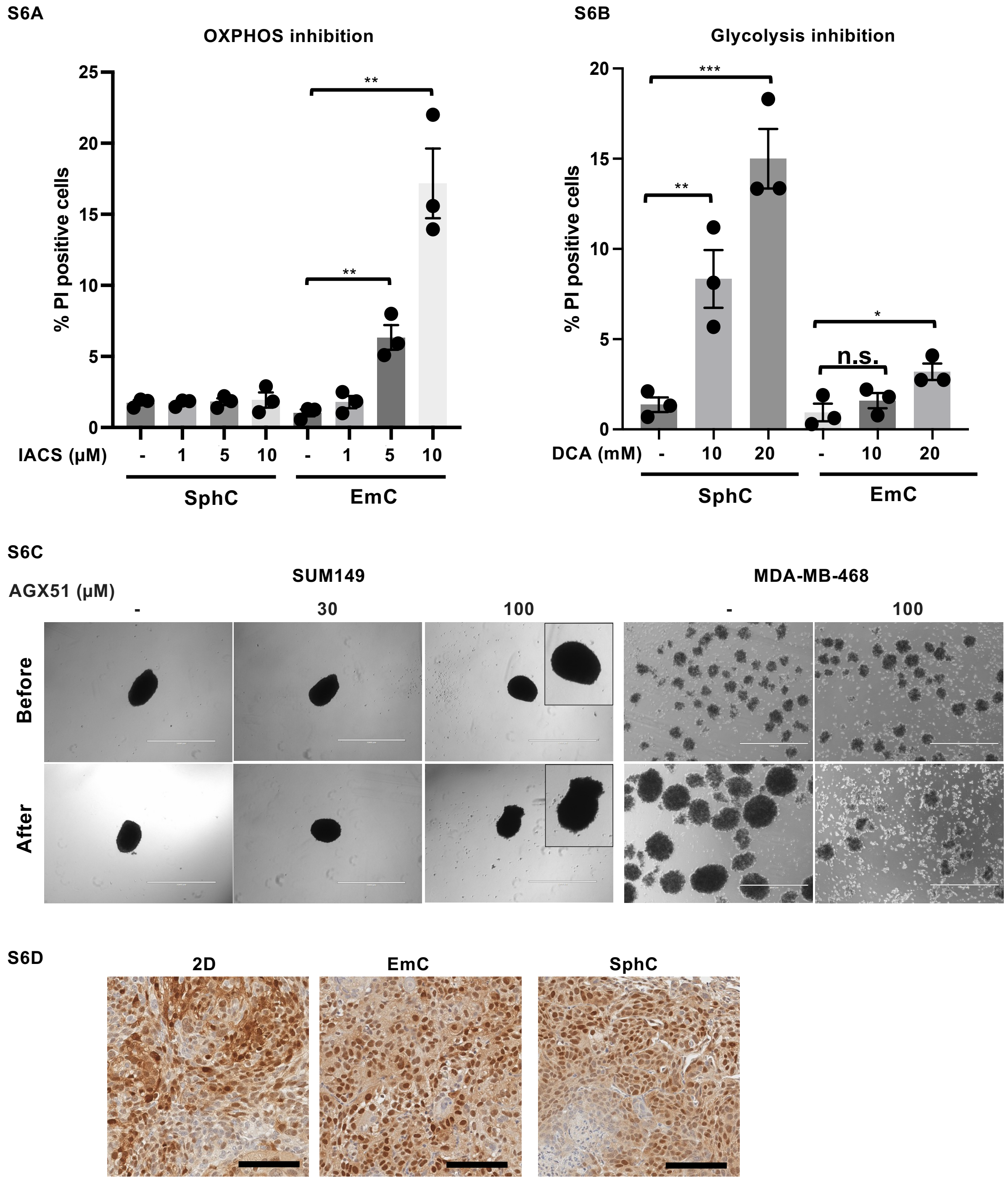

Supplementary Figure S6. Cells in EmC are sensitive to OXPHOS and ID1/ID3 inhibitors. A, Percent PI positive cells in EmC treated with different doses of IACS-010579 for 72 h. **B,** Percent PI positive cells in SphC treated with different doses of DCA for 72 h. **C,** Bright filed images of SUM149 and MDA-MB-468 cells in EmC before and after treatment with ID1/ID3 inhibitor, AGX51. Scale bar=1 mm. Inset shows high magnification to visualize fraying periphery of the embolus in the presence of AGX51 of SUM159 cells. **D,** Immunohistochemistry of ID1 expression in SUM149 lung colonies derived from cells of the indicated culture conditions (scale bar = 100 µm). Data in panels A-B are mean ± SEM,*P<0.05, **P<0.01, ***P<0.001, n.s., not significant.
